# The developmental timing of spinal touch processing alterations predicts behavioral changes in genetic mouse models of autism spectrum disorders

**DOI:** 10.1038/s41593-023-01552-9

**Published:** 2024-01-17

**Authors:** Aniqa Tasnim, Ilayda Alkislar, Richard Hakim, Josef Turecek, Amira Abdelaziz, Lauren L. Orefice, David D. Ginty

**Affiliations:** 1 Department of Neurobiology, Howard Hughes Medical Institute, Harvard Medical School, Boston, MA USA; 2 Department of Molecular Biology, Massachusetts General Hospital, Boston, MA USA; 3 Department of Genetics, Harvard Medical School, Boston, MA USA

**Keywords:** Touch receptors, Autism spectrum disorders, Sensory processing, Autism spectrum disorders, Synaptic development

## Abstract

Altered somatosensory reactivity is frequently observed among individuals with autism spectrum disorders (ASDs). Here, we report that although multiple mouse models of ASD exhibit aberrant somatosensory behaviors in adulthood, some models exhibit altered tactile reactivity as early as embryonic development, whereas in others, altered reactivity emerges later in life. Additionally, tactile overreactivity during neonatal development is associated with anxiety-like behaviors and social behavior deficits in adulthood, whereas tactile overreactivity that emerges later in life is not. The locus of circuit disruption dictates the timing of aberrant tactile behaviors, as altered feedback or presynaptic inhibition of peripheral mechanosensory neurons leads to abnormal tactile reactivity during neonatal development, whereas disruptions in feedforward inhibition in the spinal cord lead to touch reactivity alterations that manifest later in life. Thus, the developmental timing of aberrant touch processing can predict the manifestation of ASD-associated behaviors in mouse models, and differential timing of sensory disturbance onset may contribute to phenotypic diversity across individuals with ASD.

## Abstract (web summary)

Tasnim et al. show that ASD-associated genes act in different compartments of somatosensory circuits and that differences in developmental timing of ASD gene function and circuit maturation contribute to phenotypic heterogeneity across ASD models.

## Main

Sensory processing dysfunction is reported in over 94% of children, adolescents and adults with autism spectrum disorders (ASDs)^1–4^, and the latest *Diagnostic and Statistical Manual of Mental Disorders* includes atypical responses to sensory stimuli as a core diagnostic criterion for ASD^5^. Notably, 60% of individuals with ASD report tactile sensitivity difficulties^4^, with the initial appearance of atypical touch reactivity often predating ASD diagnoses^6–9^. Interacting with the world through the sense of touch, particularly at young ages, plays a central role in cognitive and social development in humans, non-human primates and rodents^10–13^, and early alterations to tactile processing and reactivity can be predictive of the emergence and severity of ASD-associated traits later in life^6,14,15^. However, autism diagnoses are elusive during infancy, in part due to high variability in ASD phenotypic presentation across individuals^16^. Defining atypical sensory processing behaviors during early development as they relate to other core features of ASD may aid in early detection, diagnosis and prognosis.

The heterogeneity in ASD etiology and behavioral outcomes necessitates a greater focus on defining neurophysiological signatures that may define subgroups in ASD^17^. Moreover, understanding neurophysiological changes in touch processing in ASD will inform the design of therapeutic approaches to treat sensory symptoms, which may in turn mitigate other ASD-related symptoms in certain individuals^12^. Recent studies in genetic models of ASD have revealed that rodents harboring mutations in the ASD-associated genes *Mecp2*, *Gabrb3*, *Shank3* and *Fmr1* exhibit overreactivity to touch stimuli^12,18–24^. Peripheral mechanosensory neurons, which convey information to the spinal cord and brainstem about tactile stimuli acting on the skin, cell-autonomously require proper expression of *Mecp2*, *Gabrb3* and *Shank3* for normal tactile reactivity in adulthood^12,23,25^. Remarkably, developmental deletion of these genes only in peripheral somatosensory neurons leads to abnormal tactile behaviors, deficits in social behaviors and increased anxiety-like behaviors in adulthood. Thus, in at least some models of ASD, somatosensory deficits that arise in the periphery during development contribute to the generation of certain ASD-associated behaviors. Furthermore, genetic ablation of these genes late in postnatal development drives tactile overreactivity in adulthood but does not lead to major changes in social or anxiety-like behaviors^12^. Therefore, intact somatosensory activity during postnatal development protects against the social and anxiety-like behavioral alterations that emerge when these genes are ablated developmentally.

Here, we sought to understand how the loci of molecular and circuit disruption in disparate ASD mouse models may predict altered touch reactivity and other ASD-related behavioral manifestations in adulthood. We found that tactile behavioral alterations are observed across mutant models in adulthood, but that disruptions to tactile reactivity emerge at different developmental times. Genetic models with dysfunction in presynaptic feedback inhibition of peripheral sensory neurons exhibit touch overreactivity perinatally and display anxiety-like and social behavior deficits in adulthood. By contrast, genetic models with disruption to axodendritic/axosomatic feedforward inhibition in spinal touch circuits have normal touch reactivity neonatally and enhanced tactile reactivity later in life. However, this elevation of tactile reactivity that manifests later in life occurs in the absence of anxiety-like and social behavioral deficits. Our findings highlight neurophysiological mechanisms that underlie susceptibility to aberrant touch processing in ASD models, and we show that early overreactivity is predictive of abnormal anxiety-like and social behaviors in adulthood. Thus, differences in the developmental timing of aberrant sensory reactivity onset may contribute to the heterogeneity of core and comorbid ASD-associated behaviors.

## Results

### ASD-related behaviors in genetic mouse models

**Fig. 1:**
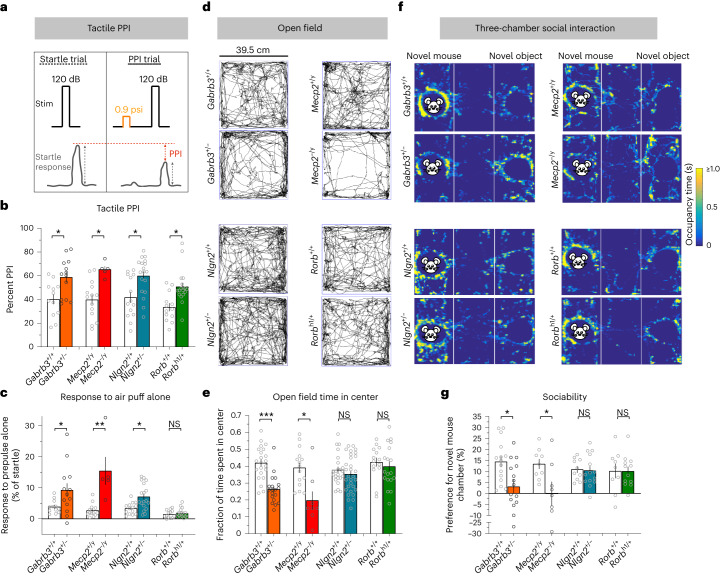
Multiple global knockout models of ASD-associated genes exhibit tactile overreactivity in adulthood, with differences in ASD-associated behaviors. **a**, Diagram showing tactile PPI of the startle reflex assay paradigm. The decrease in the startle response to a 120-dB acoustic pulse when preceded by a light air puff is defined as the effect of PPI (measured as a percentage). The interval between the prepulse and pulse was 250 ms; Stim, stimulus. **b**, Tactile PPI; *n* = 12 *Gabrb3*^+/+^ mice and *n* = 13 *Gabrb3*^+/–^ mice, *P* = 0.0071; *n* = 14 *Mecp2*^+/y^ mice and *n* = 5 *Mecp2*^–/y^ mice, *P* = 0.0033; *n* = 14 *Nlgn2*^+/+^ mice and *n* = 18 *Nlgn2*^+/–^ mice, *P* = 0.0043 for *Nlgn2*; *n* = 13 *Rorb*^+/+^ mice and *n* = 18 *Rorb*^h1/+^ mice, *P* = 0.0015. Data were analyzed by unpaired two-tailed *t*-tests. **c**, Response to the 0.9-psi air puff stimulus alone, expressed as a percentage of startle response to an acoustic startle stimulus; *n* = 12 *Gabrb3*^+/+^ mice and *n* = 13 *Gabrb3*^+/–^ mice, *P* = 0.0071; *n* = 14 *Mecp2*^+/y^ mice and *n* = 5 *Mecp2*^–/y^ mice, *P* = 0.0033; *n* = 14 *Nlgn2*^+/+^ mice and *n* = 18 *Nlgn2*^+/–^ mice, *P* = 0.0043 for *Nlgn2*; *n* = 13 *Rorb*^+/+^ mice and *n* = 18 *Rorb*^h1/+^ mice, *P* = 0.0457 for *Gabrb3*, *P* = 0.0003 for *Mecp2, P* = 0.0054 for *Nlgn2* and *P* = 0.7374 for *Rorb*. Data were analyzed by two-tailed Mann–Whitney *U*-tests. **d**, Representative activity traces in the open field assay for mutant mice and control littermates. **e**, Fraction of time spent in the center of the open field chamber; *n* = 22 *Gabrb3*^+/+^ mice and *n* = 19 *Gabrb3*^+/–^ mice, *P* < 0.0001; *n* = 15 *Mecp2*^+/y^ mice and *n* = 8 *Mecp2*^–/y^ mice, *P* = 0.0022; *n* = 23 *Nlgn2*^+/+^ mice and *n* = 30 *Nlgn2*^+/–^ mice, *P* = 0.2050 for *Nlgn2*; *n* = 14 *Rorb*^+/+^ mice and *n* = 18 *Rorb*^h1/+^ mice, *P* = 0.5339. Data were analyzed by unpaired two-tailed *t*-tests. **f**, Representative heat maps showing time spent with a novel mouse or novel object (an empty cup) in the three-chamber social interaction assay for mutant mice and control littermates. **g**, Percent preference for a chamber containing a novel mouse versus a novel object in the three-chamber social interaction assay; *n* = 15 *Gabrb3*^+/+^ mice and *n* = 15 *Gabrb3*^+/–^ mice, *P* = 0.0095; *n* = 9 *Mecp2*^+/y^ mice and *n* = 8 *Mecp2*^–/y^ mice, *P* = 0.0318; *n* = 10 *Nlgn2*^+/+^ mice and *n* = 14 *Nlgn2*^+/–^ mice, *P* = 0.8603 for *Nlgn2*; *n* = 9 *Rorb*^+/+^ mice and *n* = 12 *Rorb*^h1/+^ mice, *P* = 0.9887. Data were analyzed by unpaired two-tailed *t*-tests. For **b**, **c**, **e** and **g**, data represent mean ± s.e.m. Statistical information is provided in Supplementary Table 1; NS, not significant; *P *<* 0.05; **P < 0.001; ***P < 0.0001. Source data

**Extended Data Fig. 1:**
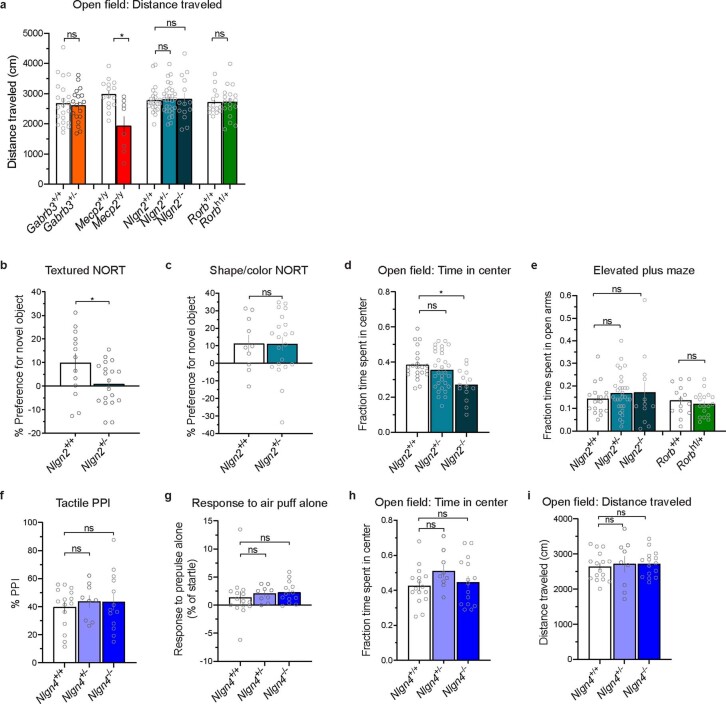
Additional behavioral characterization of global knockout ASD models. Related to Fig. 1. **a**, Distance traveled in the open field. n = 22 *Gabrb3*^+/+^ mice and n = 19 *Gabrb3*^+/-^ mice, *P* = 0.7133; n = 15 *Mecp2*^+/y^ mice and n = 8 *Mecp2*^-/y^ mice, *P* = 0.0011; n = 14 *Rorb*^+/+^ mice and n = 18 *Rorb*^h1/+^ mice, *P* = 0.9394, unpaired two-tailed t-tests. n = 23 *Nlgn2*^+/+^ mice, n = 30 *Nlgn2*^+/-^ mice, n = 14 *Nlgn2*^-/-^ mice, *P* = 0.9832 for *Nlgn2*^+/+^ vs. *Nlgn2*^+/-^, *P* = 0.9755 for *Nlgn2*^+/+^ vs. *Nlgn2*^-/-^, one-way ANOVA with post hoc Dunnett’s tests. **b, c**, Discrimination indices for textured NORT assay, n = 13 *Nlgn2*^+/+^ mice and n = 19 *Nlgn2*^+/-^ mice, *P* = 0.0376 (**b**) and shape/color NORT assay, n = 10 *Nlgn2*^+/+^ mice and n = 21 *Nlgn2*^+/-^ mice, *P* = 0.9729 (**c**), with a positive value indicating a preference for a novel object versus a familiar object. Statistical analyses were performed using unpaired two-tailed t-tests. **d**, Fraction of time spent in the center of the open field chamber. n = 23 *Nlgn2*^+/+^ mice, n = 30 *Nlgn2*^+/-^ mice, n = 14 *Nlgn2*^-/-^ mice, *P* = 0.3289 for *Nlgn2*^+/+^ vs. *Nlgn2*^+/-^, *P* = 0.0020 for *Nlgn2*^+/+^ vs. *Nlgn2*^-/-^, one-way ANOVA with post hoc Dunnett’s tests. **e**, Fraction of time spent in the open arms of the elevated plus maze. n = 17 *Nlgn2*^+/+^ mice, n = 31 *Nlgn2*^+/-^ mice, n = 12 *Nlgn2*^-/-^ mice*, P* = 0.6302 for *Nlgn2*^+/+^ vs. *Nlgn2*^+/-^, *P* = 0.6886 for *Nlgn2*^+/+^ vs. *Nlgn2*^-/-^, one-way ANOVA with post hoc Dunnett’s tests. n = 14 *Rorb*^+/+^ mice and n = 18 *Rorb*^h1/+^ mice, *P* = 0.4630 for *Rorb*, unpaired two-tailed t-test. **f**, Tactile PPI. n = 15 *Nlgn4*^+/+^ mice, n = 9 *Nlgn4*^+/-^ mice, n = 13 *Nlgn4*^-/-^ mice, *P* = 0.8054 for *Nlgn4*^+/+^ vs. *Nlgn4*^+/-^, *P* = 0.8032 for *Nlgn4*^+/+^ vs. *Nlgn4*^-/-^. **g**, Response to a 0.9 PSI air puff stimulus alone. n = 15 *Nlgn4*^+/+^ mice, n = 9 *Nlgn4*^+/-^ mice, n = 13 *Nlgn4*^-/-^ mice, *P* = 0.8100 for *Nlgn4*^+/+^ vs. *Nlgn4*^+/-^, *P* = 0.6223 for *Nlgn4*^+/+^ vs. *Nlgn4*^-/-^. **h**, Fraction of time spent in the center of the open field chamber. n = 16 *Nlgn4*^+/+^ mice, n = 9 *Nlgn4*^+/-^ mice, n = 15 *Nlgn4*^-/-^ mice, *P* = 0.1075 for *Nlgn4*^+/+^ vs. *Nlgn4*^+/-^, *P* = 0.8535 for *Nlgn4*^+/+^ vs. *Nlgn4*^-/-^. **i**, Distance traveled in the open field. n = 16 *Nlgn4*^+/+^ mice, n = 9 *Nlgn4*^+/-^ mice, n = 15 *Nlgn4*^-/-^ mice, *P* = 0.9058 for *Nlgn4*^+/+^ vs. *Nlgn4*^+/-^, *P* = 0.8561 for *Nlgn4*^+/+^ vs. *Nlgn4*^-/-^. For **a-i**, data represent means ± s.e.m. For **f-i**, statistical analyses were performed using one-way ANOVAs with post hoc Dunnett’s tests. Statistical information provided in Supplementary Table 1.

ASD-associated genes *Mecp2* and *Gabrb3* are required in peripheral somatosensory neurons for proper axoaxonic (presynaptic) feedback inhibition of these neurons and normal tactile behaviors in mice^22,23^. Consistent with previous findings, adult animals heterozygous for *Gabrb3* (*Gabrb3*^+/–^) or hemizygous for *Mecp2* (*Mecp2*^–/y^) exhibited enhanced sensitivity to back hairy skin stimulation, as measured by tactile prepulse inhibition (PPI) of an acoustic startle response and to a gentle air puff stimulus alone (Fig. 1a–c)^23^. Moreover, these animals exhibited increased anxiety-like behaviors, as measured by a reduction in time spent in the center of a chamber during the open field test (Fig. 1d,e and Extended Data Fig. 1a). *Gabrb3*^+/–^ and *Mecp2*^–/y^ animals also exhibited abnormal sociability, as measured by the time spent in a chamber containing a novel mouse compared to the time spent in a chamber containing an empty cup in the three-chamber social interaction test^23,24,26–28^ (Fig. 1f,g). To explore conserved and additional mechanisms of altered sensory reactivity in mouse models of ASD, we asked whether other ASD-associated genes implicated in inhibitory synaptic signaling are also required for tactile behaviors.

The ASD-associated genes *Nlgn2* and *Nlgn4* encode the synaptic adhesion molecules neuroligin 2 (NLGN2) and neuroligin 4 (NLGN4), respectively, which contribute to GABA_A_ and glycine receptor synapse function in the forebrain and brainstem^29–32^. We observed that animals harboring loss-of-function mutations in *Nlgn2*^33^ exhibited increased responsivity to hairy skin stimulation (Fig. 1b,c). Additionally, *Nlgn2*^+/–^ animals displayed deficits in a texture discrimination assay but exhibited normal preferences for novel objects differing in shape and color, indicating intact novelty preference (Extended Data Fig. 1b,c). Despite these marked changes in tactile reactivity and texture discrimination, *Nlgn2*^+/–^ mice showed no alterations in anxiety-like or social interaction behaviors compared to control littermates (Fig. 1d–g and Extended Data Fig. 1a,d,e), although *Nlgn2*^–/–^ mice exhibited reduced time spent in the center of the open field (Extended Data Fig. 1a,c,d). By contrast, *Nlgn4*-mutant animals^34^ did not exhibit detectable tactile or anxiety-like behavioral alterations (Extended Data Fig. 1f–i), and prior studies observed intact social behaviors in these mutant animals^35^, which may be consistent with significant evolutionary divergence of *Nlgn4* in the mouse genome from other mammalian NLGN4 genes^36,37^.

We also examined the ASD-associated gene *Rorb*^38^, which is expressed by a population of spinal cord interneurons that mediate axodendritic/axosomatic feedforward inhibition of spinal projection neurons in the deep dorsal horn^39,40^. The activity of *Rorb*-expressing interneurons is required for normal tactile and sensorimotor function^39,41^. To investigate the role of the *Rorb* gene in ASD-associated behaviors, we tested animals with a pathogenic mutation arising at the *Rorb* locus (*Rorb*^h1^)^42,43^. As observed in *Gabrb3*^+/–^, *Mecp2*^–/y^ and *Nlgn2*^+/–^ animals, *Rorb*^h1/+^ mice exhibited enhanced tactile sensitivity in the tactile PPI assay, although *Rorb*^h1/+^ mice did not have enhanced reactivity to a gentle air puff alone (Fig. 1b,c). Like *Nlgn2*^+/–^ animals, *Rorb*-mutant mice showed normal anxiety-like and social behaviors compared to control littermates (Fig. 1d–g and Extended Data Fig. 1a,e). Together, these findings reveal considerable heterogeneity across mouse models of ASD in adulthood; although *Gabrb3*^+/–^, *Mecp2*^–/y^, *Nlgn2*^+/–^ and *Rorb*^h1/+^ animals all exhibit aberrant tactile behaviors, *Gabrb3*^+/–^ and *Mecp2*^–/y^ animals also display enhanced anxiety-like behaviors and reduced sociability^23^, whereas *Nlgn2*^+/–^ and *Rorb*^h1/+^ animals do not.

### Emergence of tactile overreactivity in development

Our results demonstrate that while tactile overreactivity is a characteristic shared by multiple ASD mouse models in adulthood, there are differences in comorbid ASD-like behaviors. Our prior work in *Gabrb3*, *Mecp2* and *Shank3* ASD mouse models revealed a developmental window before postnatal day 10 (P10) during which normal tactile sensitivity is required for the formation of normal brain microcircuit properties and certain cognitive and social behaviors^12,23^. Expression of these genes in somatosensory neurons of the dorsal root ganglia (DRG) was required during early postnatal development for normal tactile, anxiety-like and social behaviors in adulthood. Thus, we hypothesized that tactile overreactivity during neonatal and early postnatal life may determine the extent to which mice display altered anxiety-like and social behaviors later in adulthood.

**Fig. 2:**
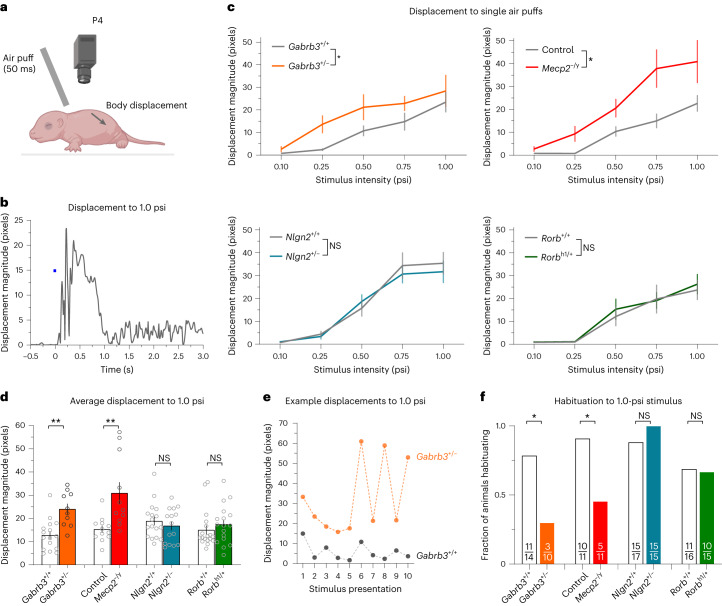
Neonatal tactile reactivity in global knockout ASD mouse models. **a**, Diagram showing the experimental paradigm for the neonatal air puff responsivity assay. Air puff tubing was affixed 3 mm above the nape of the neck of neonatal mice. Maximal body displacements in the 500 ms following a 50-ms air puff were measured using optic flow analysis. Parts were created with BioRender.com. **b**, Example displacement trace of a control P4 mouse to a single 1.0-psi air puff stimulus. **c**, Displacement responses to a single presentation of 0.10-, 0.25-, 0.50-, 0.75- and 1.0-psi stimuli; *n* = 16 *Gabrb3*^+/+^ mice and *n* = 10 *Gabrb3*^+/–^ mice; *n* = 12 *Mecp2*^+/y^ mice and *n* = 11 *Mecp2*^–/y^ mice; *n* = 17 *Nlgn2*^+/+^ mice and *n* = 15 *Nlgn2*^+/–^ mice; *n* = 18 *Rorb*^+/+^ mice and *n* = 17 *Rorb*^h1/+^ mice. Data were analyzed by mixed-effects analyses, effect of genotype; *Gabrb3*: *F*_1,24_ = 9.060 and *P* = 0.0030; *Mecp2*: *F*_1,21_ = 9.253 and *P* = 0.0062; *Nlgn2*: *F*_1,30_ = 0.1071 and *P* = 0.7457; *Rorb*: *F*_1,33_ = 0.1204 and *P* = 0.7308. Data are shown as mean ± s.e.m. **d**, Average displacement responses to ten presentations of a 1.0-psi stimulus at 20- to 30-s interstimulus intervals; *n* = 16 *Gabrb3*^+/+^ mice and *n* = 10 *Gabrb3*^+/–^ mice; *n* = 12 *Mecp2*^+/y^ mice and *n* = 11 *Mecp2*^–/y^ mice; *n* = 17 *Nlgn2*^+/+^ mice and *n* = 15 *Nlgn2*^+/–^ mice; *n* = 18 *Rorb*^+/+^ mice and *n* = 17 *Rorb*^h1/+^ mice. *P* = 0.0003 for *Gabrb3*, *P* = 0.0018 for *Mecp2*, *P* = 0.2258 for *Nlgn2* and *P* = 0.1970 for *Rorb*. Data were analyzed by unpaired one-tailed *t*-tests and are shown as mean ± s.e.m. **e**, Example traces showing displacements to ten presentations of 1.0-psi stimuli from a *Gabrb3*^+/+^ and *Gabrb3*^+/–^ mouse. To calculate habituation, the average of the last three stimulus presentation responses was compared to the average of the first three responses. A >25% reduction in responsivity between blocks was considered to be a habituation response. **f**, Fraction of animals habituating to repeated presentations of 1.0-psi air puffs; *P* = 0.0244 for *Gabrb3, P* = 0.0317 for *Mecp2*; *P* = 0.7258 for *Nlgn2* and *P* = 0.6017 for *Rorb*. Data were analyzed by one-sided Fisher’s exact tests. Statistical information is provided in Supplementary Table 1; *P *<* 0.05; **P < 0.001. Source data

**Extended Data Fig. 2:**
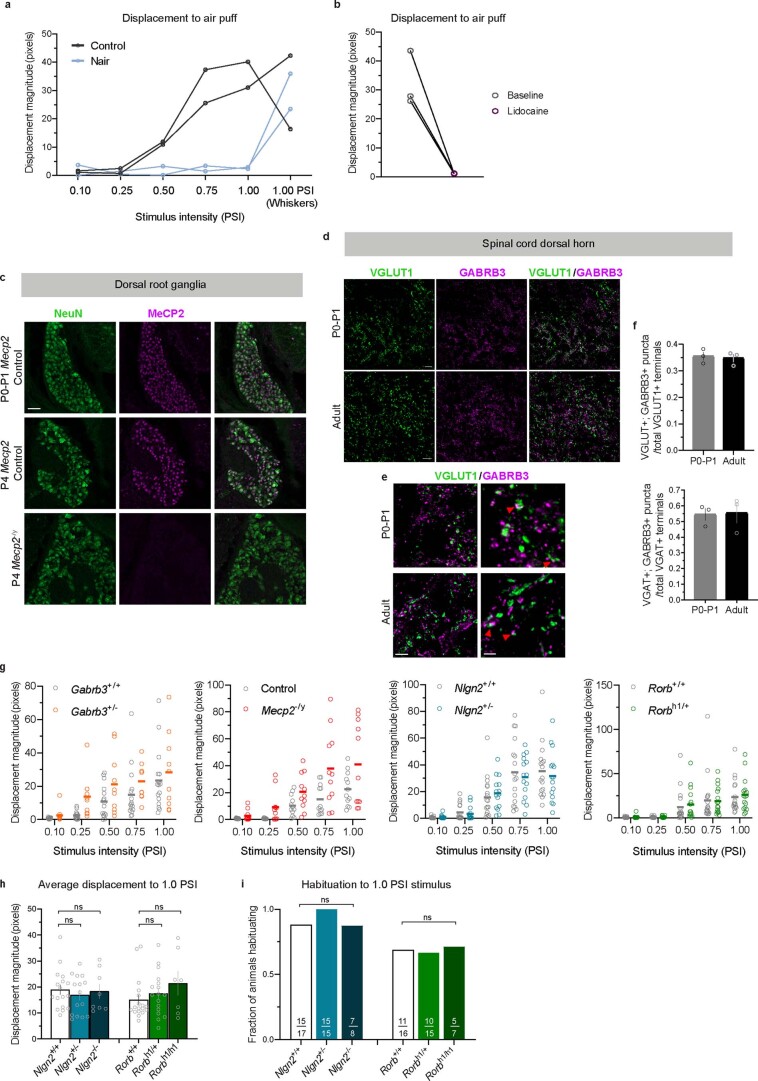
Additional behavioral and molecular characterization of ASD mouse models during neonatal ages. Related to Fig. 2. **a**, Displacement responses to single air puff stimuli of varying intensities delivered in sequence to the nape of the neck, in P4 animals treated with Nair on the back hairy skin and non-treated animals. At the end of back hairy skin trials, the 1.0 PSI stimulus was delivered to the whisker pad. N = 2 control mice and N = 2 Nair-treated mice. **b**, Displacement responses to a single 1.0 PSI air puff stimulus before and after topical lidocaine application to the nape of the neck. Each pair of dots represents the same P4 animal pre- and post-application. N = 3 mice. **c**, Dorsal root ganglia immunohistochemistry showing expression of NeuN and MeCP2 in control mice at P0-P1 and P4, and in *Mecp2*^-/y^ mice to validate antibody specificity. Scale bar denotes 50 µm. **d**-**e**, Spinal cord immunohistochemistry showing expression of VGLUT1-labeled synaptic terminals and GABRB3 in control P0-P1 and adult mice. Scale bars denote 10 µm (**d**), 5 µm (**e**, left column), and 2 µm (**e**, right column). Red arrows denote co-labeled puncta. **f**, Quantification of VGLUT1 and GABRB3 (top) or VGAT and GABRB3 (bottom) co-labeled puncta, as a fraction of total VGLUT1+ or VGAT+ puncta in a field of view, at P0-P1 or adulthood. Red arrows denote examples of proximity between markers. Each dot represents the average value for one animal, with three spinal cord images taken per animal. N = 3 animals for both conditions. **g**, Displacement responses to a single presentation of 0.10, 0.25, 0.50, 0.75 and 1.0 PSI stimuli for global mutant animals. Each dot represents the displacement response of a single animal on an air puff trial, and dashes represent means. n = 16 *Gabrb3*^+/+^ mice and n = 10 *Gabrb3*^+/-^ mice; n = 12 *Mecp2*^+/y^ mice and n = 11 *Mecp2*^-/y^ mice; n = 17 *Nlgn2*^+/+^ mice and n = 15 *Nlgn2*^+/-^ mice; n = 18 *Rorb*^+/+^ mice and n = 17 *Rorb*^h1/+^ mice. **h**, Average displacement responses to 10 presentations of a 1.0 PSI stimulus at 20-30 second interstimulus intervals. n = 17 *Nlgn2*^+/+^ mice, n = 15 *Nlgn2*^+/-^ mice, n = 8 *Nlgn2*^-/-^ mice, *P* = 0.6848 for *Nlgn2*^+/+^ vs. *Nlgn2*^+/-^, *P* = 0.9749 for *Nlgn2*^+/+^ vs. *Nlgn2*^-/-^. n = 18 *Rorb*^+/+^ mice, n = 17 *Rorb*^h1/+^ mice, n = 7 *Rorb*^h1/h1^ mice, *P* = 0.6596 for *Rorb*^+/+^ vs. *Rorb*^h1/+^, *P* = 0.2211 for *Rorb*^+/+^ vs. *Rorb*^h1/h1^, one-way ANOVAs with post hoc Dunnett’s tests. **i**, Fraction of animals habituating to repeated presentations of 1.0 PSI air puffs. *P* = 0.7043 for *Nlgn2*^+/+^ vs. *Nlgn2*^-/-^, *P* = 0.6495 for *Rorb*^+/+^ vs. *Rorb*^h1/h1^, one-sided Fisher’s Exact tests. For **a**, **f**, and **h**, data represent means ± s.e.m. Statistical information provided in Supplementary Table 1.

We asked when during development tactile overreactivity emerges in the *Gabrb3*^+/–^, *Mecp2*^–/y^, *Nlgn2*^+/–^ and *Rorb*^h1/+^ mouse models. To address this, we assessed reactivity to light air puff stimuli applied to the back hairy skin of early postnatal age mice. We chose a time point within the first postnatal week, P4, to measure air puff reactivity. P4 was ideal for these measurements because we observed substantial hair shaft emergence across strains compared to younger pups and relative quiescence on an experimental platform during interstimulus intervals compared to older pups. Air puffs delivered to the back hairy skin of P4 pups resulted in body displacement responses, and we measured displacement of the back region of the mouse to quantify movement (Fig. 2a,b and Supplementary Video 1). Given the lack of clearly identifiable visual features in this region, we turned to optical flow point tracking, which provided high accuracy measurements of animal displacement. In this assay, responsivity increased as the air puff stimulus intensity increased, was dependent on hair deflection (Extended Data Fig. 2a) and was lost following topical application of lidocaine to the back hairy skin to silence cutaneous sensory nerve fibers (Extended Data Fig. 2b).

We hypothesized that *Gabrb3*^+/–^ and *Mecp2*^–/y^ animals, which exhibit enhanced anxiety-like behaviors and sociability deficits in adulthood, would display aberrant tactile reactivity at this postnatal time point. In our experimental paradigm, we assessed reactivity to single presentations of 0.10-, 0.25-, 0.50- and 0.75-psi air puff stimuli, followed by ten presentations of a 1.0-psi stimulus at 20- to 30-s intervals. Consistent with our hypothesis, P4 *Gabrb3*^+/–^ and *Mecp2*^–/y^ animals exhibited significantly enhanced reactivity to the individual presentations of the range of stimuli (Fig. 2c and Extended Data Fig. 2g) as well as to the repeated presentations of the 1.0-psi stimulus (Fig. 2d). Additionally, we assessed habituation to the repeated 1.0-psi air puff stimulus, as a lack of sensory habituation to repeated stimuli, including in the tactile domain, is frequently observed in individuals with or at high risk of ASD diagnoses^44–47^. To do so, we measured the decrease in response between the averages of the first and last three presentations of the 1.0-psi stimulus (Fig. 2e) and categorized animals as habituating if they showed a >25% decrease in responsivity between these blocks. Interestingly, although the majority of control P4 pups exhibited habituation to repeated stimuli, smaller proportions of *Gabrb3*^+/–^ and *Mecp2*^–/y^ animals exhibited habituation (Fig. 2f). Using immunohistochemical analyses, we confirmed that MECP2 and GABRB3 are indeed expressed perinatally at the protein level in the DRG and near axon terminals of DRG neurons, respectively (Extended Data Fig. 2c–f).

By contrast, and consistent with the hypothesis that normal tactile reactivity during development may be predictive of normal anxiety-like and social behaviors in adulthood, the reactivity of *Nlgn2*- and *Rorb*-mutant animals to air puffs was indistinguishable from that of control littermates at P4 (Fig. 2c,d and Extended Data Fig. 2g,h). Moreover, these animals showed habituation that was comparable to that in control littermates (Fig. 2f and Extended Data Fig. 2i). Thus, aberrant tactile behaviors emerge at different times across the ASD mouse models tested here. Although *Nlgn2*^+/–^, *Rorb*^h1/+^, *Gabrb3*^+/–^ and *Mecp2*^–/y^ animals all display tactile overreactivity in adulthood, only *Gabrb3*^+/–^ and *Mecp2*^–/y^ animals exhibit tactile overreactivity at P4. *Nlgn2*^+/–^ and *Rorb*^h1/+^ animals are behaviorally indistinguishable from control littermates at this early postnatal time but exhibit aberrant tactile behaviors in adulthood.

### Embryonic tactile overreactivity

**Fig. 3:**
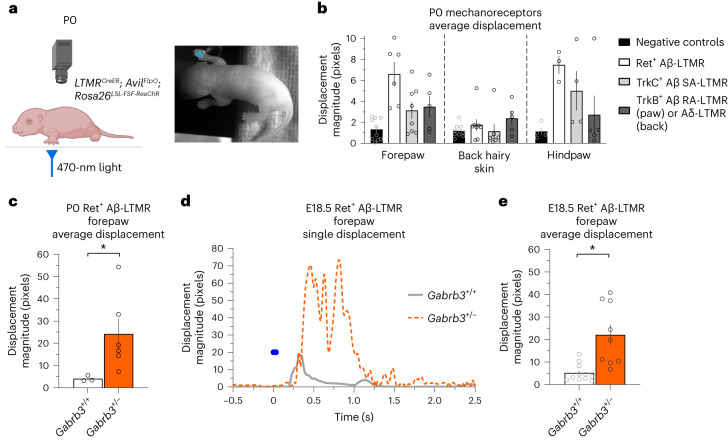
*Gabrb3*-mutant mice are overreactive to activation of Aβ-LTMRs at birth and E18.5. **a**, Experimental setup for the perinatal mechanoreceptor optical activation assay. P0 or E18.5 mice were placed on a clear acrylic stage, and LED illumination was directed to the paw or back hairy skin. Parts created with BioRender.com. **b**, Average displacement responses to five optical stimuli (20- to 30-s interstimulus intervals) delivered to mechanoreceptor subtypes in the forepaw, back hairy skin and hindpaw compared to control littermates that lack opsin expression (controls include opsin^+^Cre^–^ and opsin^–^Cre^+^ animals). For the forepaw, *n* = 10 negative-control mice, *n* = 6 Ret^+^ mice, *n* = 8 TrkC^+^ mice and *n* = 5 TrkB^+^ mice. For back hairy skin, *n* = 9 negative-control mice, *n* = 8 Ret^+^ mice, *n* = 7 TrkC^+^ mice and *n* = 5 TrkB^+^ mice. For the hindpaw, *n* = 4 negative-control mice, *n* = 3 Ret^+^ mice, *n* = 4 TrkC^+^ mice and *n* = 5 TrkB^+^ mice. SA-LTMR, slowly adapting LTMR; RA-LTMR, rapidly adapting LTMR. **c**, Average displacement responses to five optical stimuli activating Ret^+^ Aβ-LTMRs to the forepaws of P0 *Ret*^*creER*^; *Avil*^*FlpO*^; *Rosa26*^*LSL-FSF-ReaChR-mCitrine*^; *Gabrb3*^+/+^ and *Gabrb3*^+/–^ animals; *n* = 3 *Gabrb3*^+/+^ mice and *n* = 6 *Gabrb3*^+/–^ mice; *P* = 0.0169. Data were analyzed by unpaired one-tailed Welch’s *t*-test. **d**, Example displacement traces from an E18.5 *Gabrb3*^+/+^ mouse and *Gabrb3*^+/–^ mouse to optical activation of Ret^+^ Aβ-LTMRs in the forepaw. **e**, Average displacement responses to five optical stimuli activating Ret^+^ Aβ-LTMRs in the forepaws of E18.5 control and mutant animals; *n* = 11 *Gabrb3*^+/+^ mice and *n* = 9 *Gabrb3*^+/–^ mice; *P* = 0.0029. Data were analyzed by unpaired one-tailed Welch’s *t*-test. For **b**, **c** and **e**, data are shown as mean ± s.e.m. Statistical information is provided in Supplementary Table 1; *P < 0.05. Source data

Because a subset of ASD mutant models displayed tactile overreactivity at P4, we next asked whether aberrant tactile behavior emerges even earlier in development. Additionally, we sought to test whether selective activation of mechanosensory neuron subtypes that mediate light touch would be sufficient to promote aberrant behavioral reactivity in mutant animals. To test this, we used optogenetic activation of low-threshold mechanoreceptor (LTMR) terminals in the skin and assessed the extent of behavioral reactivity to LTMR subtype-specific stimulation in P0 mice (Fig. 3a).

**Extended Data Fig. 3:**
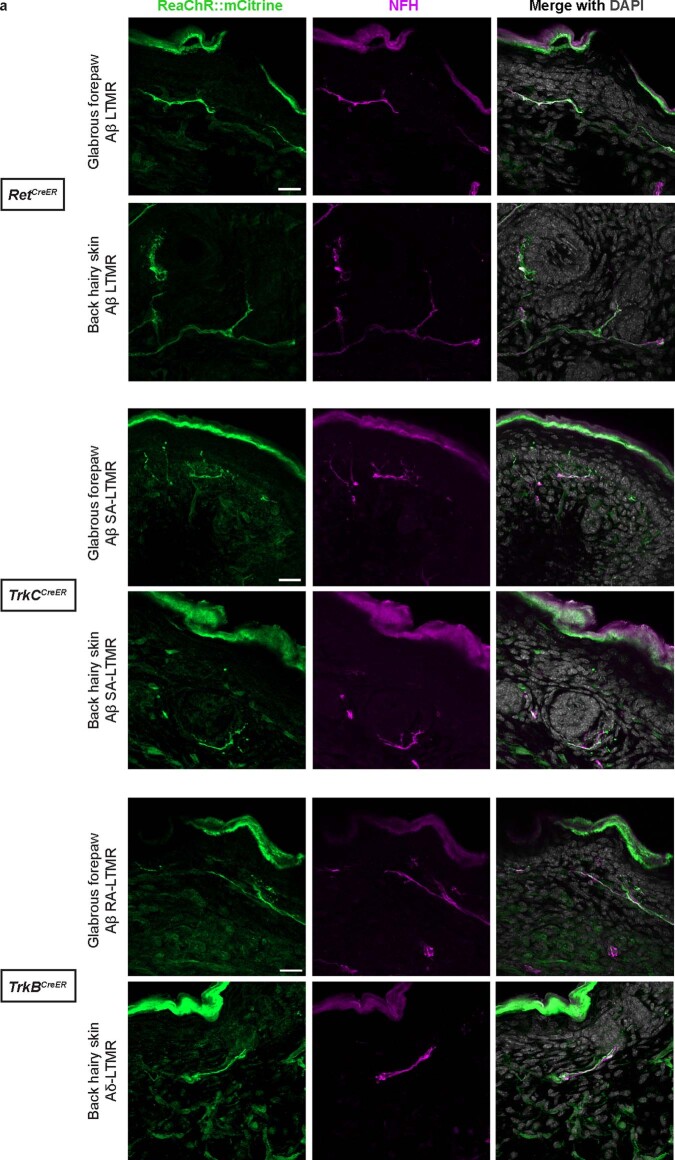
Anatomical characterization of opsin expression in low-threshold mechanoreceptor labeling paradigm at birth. Related to Fig. 3. **a**, Glabrous forepaw and back hairy skin immunohistochemistry showing expression of ReaChR::mCitrine (green), Neurofilament Heavy Chain (NFH; magenta), and DAPI (gray) from P0 animals with *Ret*^*CreER*^, *TrkC*^*CreER*^, or *TrkB*^*CreER*^ mechanosensory neuron labeling strategies. Scale bars denote 20 µm.

Using a Cre- and Flp-dependent ReaChR optogenetic actuator line, we first asked whether control P0 pups reacted to activation of large-diameter LTMRs that terminate in the forepaw, hindpaw and back hairy skin. Using the intersection of *Avil*^*FlpO*^ (to label all peripheral sensory neurons)^48^ and LTMR-specific CreER (LTMR^CreER^) lines, we targeted Ret^+^ fast-conducting LTMRs (Aβ-LTMRs; using *Ret*^*creER*^)^49^, slowly adapting LTMRs (using *TrkC*^*creER*^)^50^ and rapidly adapting LTMRs (in the glabrous paw and Aδ-LTMRs in the hairy skin; using *TrkB*^*creER*^^51^) (Fig. 3b and Extended Data Fig. 3a). At this P0 time point, large-diameter sensory neurons, labeled by neurofilament heavy chain (NFH), expressed ReaChR::mCitrine and innervated both glabrous and hairy skin (Extended Data Fig. 3a). We measured reactivity to stimulation using a 470-nm light pulse delivered five times at 20- to 30-s interstimulus intervals to the forepaws, back hairy skin and hindpaws of P0 animals. We found that paw Ret^+^ Aβ-LTMR stimulation evoked the greatest degree of reactivity (Fig. 3b). In contrast to behavioral responses evoked from the paw regions, activation of these same populations in back hairy skin resulted in less robust responses. We next tested forepaw activation of Ret^+^ Aβ-LTMRs in *Gabrb3* mutants and their control littermates and found that mutant animals displayed enhanced responses to stimulation of Ret^+^ Aβ-LTMRs at P0 (Fig. 3c). We also tested animals just before birth on embryonic day 18.5 (E18.5). Reactivity could be reliably evoked from control E18.5 animals, and, remarkably, the *Gabrb3* mutants were significantly more reactive to forepaw optical stimulation (Fig. 3d,e and Supplementary Video 2), revealing a role for this ASD-associated gene in controlling tactile reactivity at late embryonic stages. Thus, selective activation of a subset of LTMRs innervating the paw is sufficient to evoke behavioral responses in E18.5 and P0 mice, and loss of function of *Gabrb3* can drive tactile behavioral overreactivity at these ages.

### Distinct loci of function for *Gabrb3* and *Nlgn2* in behavior

**Fig. 4:**
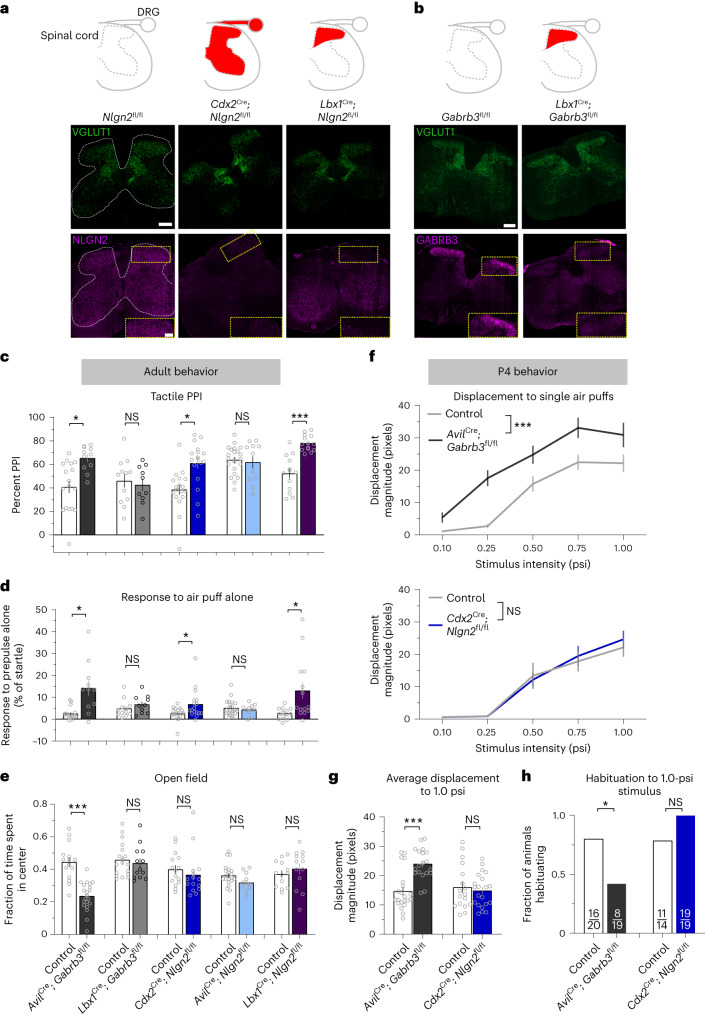
Differential cell-autonomous requirements for *Gabrb3* and *Nlgn2* in peripheral and spinal cord neuron types for tactile behaviors. **a**,**b**, Schematics showing areas of Cre activity (shaded in red) in the spinal cord and/or DRG for the genetic strategies shown below (top row). Immunohistochemistry of the adult spinal cord shows the expression of VGLUT1-labeled synaptic terminals and NLGN2 in control, *Cdx2*^Cre^; *Nlgn2*^fl/fl^ and *Lbx1*^Cre^; *Nlgn2*^fl/fl^ mice (**a**) and expression of VGLUT and GABRB3 in control and *Lbx1*^Cre^; *Gabrb3*^fl/fl^ mice (**b**). White dotted lines show the outline of the spinal cord gray matter, and yellow dotted boxes and insets show the dorsal horn; scale bars, 200 µm (full-size images) and 100 µm (insets). **c**, Tactile PPI; *n* = 17 control and *n* = 11 *Avil*^Cre^; *Gabrb3*^fl/fl^ mice, *P* = 0.0014; *n* = 12 control and *n* = 10 *Lbx1*^Cre^; *Gabrb3*^fl/fl^ mice, *P* = 0.6746; *n* = 18 control and *n* = 17 *Cdx2*^Cre^; *Nlgn2*^fl/fl^ mice, *P* = 0.0010; *n* = 22 control and *n* = 11 *Avil*^Cre^; *Nlgn2*^fl/fl^ mice, *P* = 0.7051; *n* = 13 control and *n* = 14 *Lbx1*^Cre^; *Nlgn2*^fl/fl^ mice, *P* < 0.0001. Data were analyzed by unpaired two-tailed *t*-tests. **d**, Response to a 0.9-psi air puff stimulus alone; *n* = 17 control and *n* = 11 *Avil*^Cre^; *Gabrb3*^fl/fl^ mice, *P* = 0.0014; *n* = 12 control and *n* = 10 *Lbx1*^Cre^; *Gabrb3*^fl/fl^ mice, *P* = 0.6746; *n* = 18 control and *n* = 17 *Cdx2*^Cre^; *Nlgn2*^fl/fl^ mice, *P* = 0.0010; *n* = 22 control and *n* = 11 *Avil*^Cre^; *Nlgn2*^fl/fl^ mice, *P* = 0.7051; *n* = 13 control and *n* = 14 *Lbx1*^Cre^; *Nlgn2*^fl/fl^ mice; *P* = 0.0011 for *Avil*^Cre^; *Gabrb3*^fl^; *P* = 0.2543 for *Lbx1*^Cre^; *Gabrb3*^fl^; *P* = 0.0204 for *Cdx2*^Cre^; *Nlgn2*^fl^; *P* = 0.9919 for *Avil*^Cre^; *Nlgn2*^fl^; *P* = 0.0107 for *Lbx1*^Cre^; *Nlgn2*^fl^. Data were analyzed by two-tailed Mann–Whitney *U*-tests. **e**, Fraction of time spent in the center of the open field chamber; *n* = 15 control and *n* = 21 *Avil*^Cre^; *Gabrb3*^fl/fl^ mice*, P* < 0.0001; *n* = 18 control and *n* = 13 *Lbx1*^Cre^; *Gabrb3*^fl/fl^ mice, *P* = 0.4169; *n* = 15 control and *n* = 15 *Cdx2*^Cre^; *Nlgn2*^fl/fl^ mice, *P* = 0.4524; *n* = 22 control and *n* = 11 *Avil*^Cre^; *Nlgn2*^fl/fl^ mice, *P* = 0.1019; *n* = 13 control and *n* = 13 *Lbx1*^Cre^; *Nlgn2*^fl/fl^ mice, *P* = 0.4214. Data were analyzed by unpaired two-tailed *t*-tests. **f**, Displacement response to a single presentation of 0.10-, 0.25-, 0.50-, 0.75- and 1.0-psi stimuli at P4; *n* = 21 control and *n* = 19 *Avil*^Cre^; *Gabrb3*^fl/fl^ mice; *n* = 17 control and *n* = 21 *Cdx2*^Cre^; *Nlgn2*^fl/fl^ mice. Data were analyzed by mixed-effects analyses, effect of genotype; *Avil*^Cre^; *Gabrb3*^fl^: *F*_1,38_ = 36.38, *P* < 0.0001; *Cdx2*^Cre^; *Nlgn2*^fl^: *F*_1,36_ = 0.1169, *P* = 0.7344. **g**, Average displacement responses to ten presentations of a 1.0-psi stimulus at 20- to 30-s interstimulus intervals; *n* = 21 control and *n* = 19 *Avil*^Cre^; *Gabrb3*^fl/fl^ mice; *n* = 17 control and *n* = 21 *Cdx2*^Cre^; *Nlgn2*^fl/fl^ mice. *P* < 0.0001 for *Avil*^Cre^; *Gabrb3*^fl^; *P* = 0.2382 for *Cdx2*^Cre^; *Nlgn2*^fl^. Data were analyzed by unpaired one-tailed *t*-tests. **h**, Fraction of animals habituating to repeated presentations of 1.0-psi air puffs. For *Gabrb3*, *P* = 0.0171, and for *Nlgn2*, *P* = 0.9333. Data were analyzed by one-sided Fisher’s exact tests. For **c**–**g**, data are shown as mean ± s.e.m. Statistical information is provided in Supplementary Table 1; *P *<* 0.05; ***P < 0.0001. Source data

**Extended Data Fig. 4:**
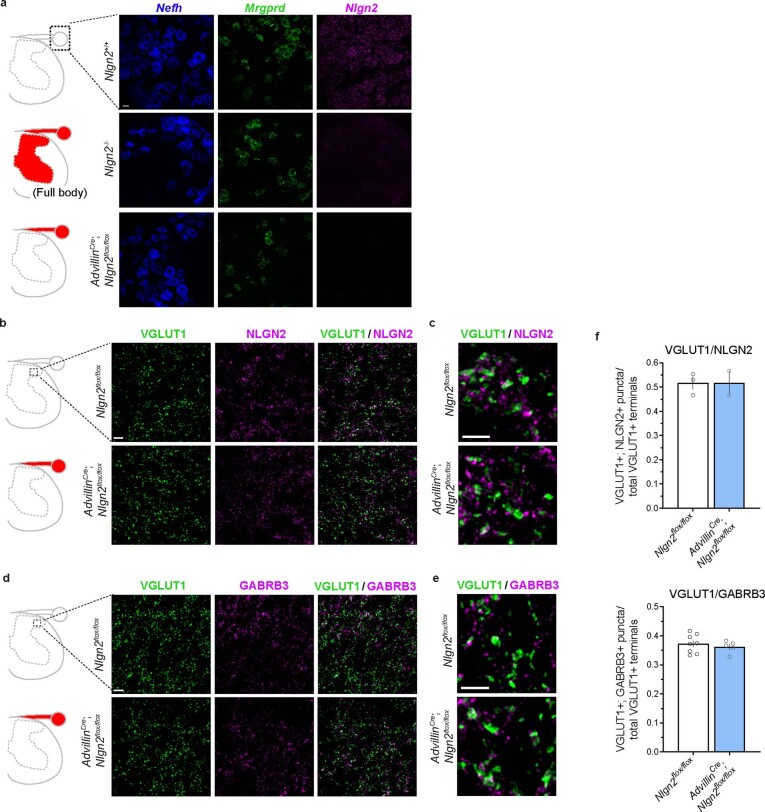
Immunohistochemical characterization of *Nlgn2* sensory neuron knockout tissue. Related to Fig. 4. **a**, Schematics showing imaging area (black dotted box) and areas of Cre activity, where relevant (shaded in red) (left). *In situ* hybridization labeling using RNAScope for *Nefh* (large-diameter DRG neurons), *Mrgprd* (small-diameter nonpeptidergic DRG neurons), and *Nlgn2* transcripts in DRG cells of *Nlgn2*^+/+^, *Nlgn2*^+/-^, and *Advillin*^*Cre*^; *Nlgn2*^*flox/flox*^ mice (right). Scale bar denotes 20 µm. **b-e**, Spinal cord immunohistochemistry showing expression of VGLUT1-labeled synaptic terminals and NLGN2 (**b, c**) and GABRB3 (**d, e**) in control and *Advillin*^*Cre*^; *Nlgn2*^*flox/flox*^ mice. Scale bars denote 10 µm (**b, d**) and 5 µm (**c, e**). **f**, Quantification of VGLUT1 and NLGN2 (top) and VGLUT1 and GABRB3 (bottom) co-labeled puncta, as a fraction of total VGLUT1+ puncta in a field of view. Each dot represents the average value for one animal, with three spinal cord images taken per animal. N = 3 *Nlgn2*^*flox/flox*^ animals and N = 2 *Advillin*^*Cre*^; *Nlgn2*^*flox/flox*^ mice (top) and N = 8 *Nlgn2*^*flox/flox*^ animals and N = 5 *Advillin*^*Cre*^; *Nlgn2*^*flox/flox*^ mice (bottom). Data represent means ± s.e.m.

Axoaxonic feedback inhibition, or presynaptic inhibition, is mediated by GABA_A_ receptors (GABA_A_Rs) present on peripheral somatosensory neuron afferent fibers, and deletion of *Gabrb3* or *Mecp2* in these neurons leads to a loss of GABA_A_R-mediated feedback inhibition. Consistent with a role in organizing both GABAergic and glycinergic synapses, the gene product of *Nlgn2* was found to be required for both types of inhibitory neurotransmission in the medulla^30^. Interestingly, NLGN2 is found in native GABA_A_R complexes in the mouse brain^52^, and its transcripts are expressed in DRG neurons (Extended Data Fig. 4a). Therefore, we tested whether NLGN2 is involved in GABA_A_R scaffolding in sensory axon terminals. Both NLGN2 and GABA_A_R immunoreactivity associated with vesicular glutamate transporter 1-positive (VGLUT1^+^) sensory axon terminals in the dorsal horn of mice lacking NLGN2 in sensory neurons (*Avil*^Cre^; *Nlgn2*^fl/fl^ mice) was normal (Extended Data Fig. 4b–f), despite complete loss of *Nlgn2* expression in the DRGs of these mutants (Extended Data Fig. 4a). Thus, unlike GABRB3 and MECP2, NLGN2 is not required in peripheral somatosensory neurons for GABA_A_R localization to presynaptic terminals. Additionally, animals with either *Gabrb3* or *Mecp2* deleted from peripheral somatosensory neurons exhibited tactile overreactivity in the tactile PPI assay and in response to an air puff alone^22,23^ (Fig. 1b,c), which was not the case in *Avil*^Cre^; *Nlgn2*^fl/fl^ mice (Fig. 4c,d), indicating that *Nlgn2* expression is not required in somatosensory neurons for tactile sensitivity.

**Extended Data Fig. 5:**
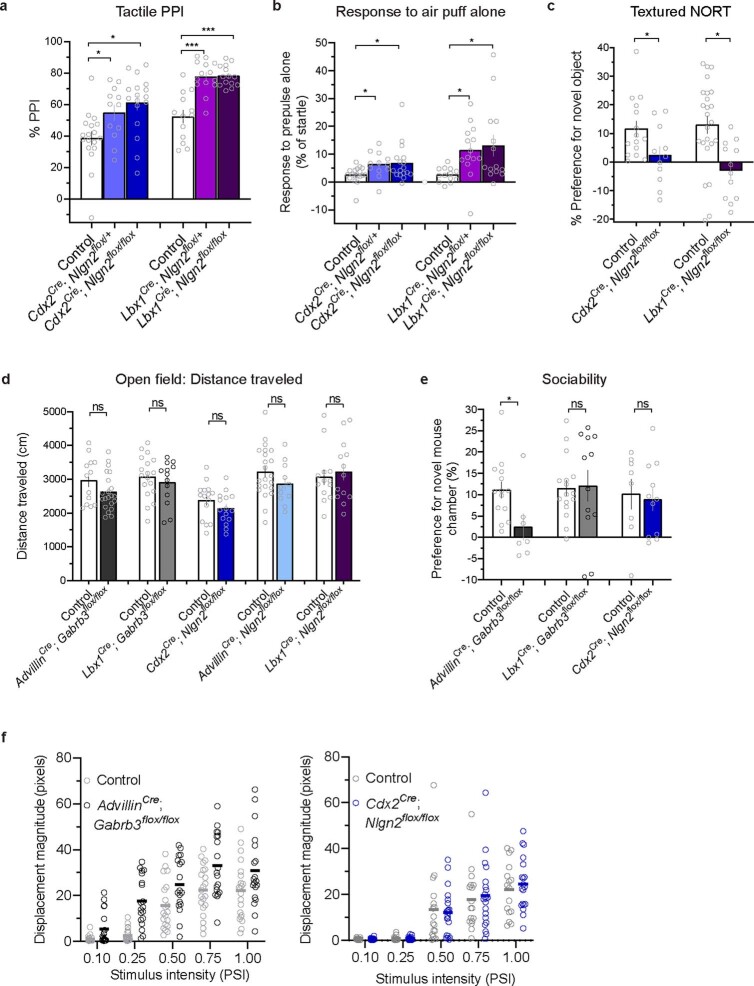
Additional behavioral characterization of conditional knockout ASD models. Related to Fig. 4. **a**, Tactile PPI. n = 18 control mice, n = 12 *Cdx2*^*Cre*^; *Nlgn2*^*flox/+*^ mice, n = 17 *Cdx2*^*Cre*^; *Nlgn2*^*flox/flox*^ mice, *P* = 0.0367 for Control vs. *Cdx2*^*Cre*^; *Nlgn2*^*flox/+*^, *P* = 0.0012 for Control vs. *Cdx2*^*Cre*^; *Nlgn2*^*flox/flox*^. n = 13 control mice, n = 14 *Lbx1*^*Cre*^; *Nlgn2*^*flox/+*^ mice, n = 14 *Lbx1*^*Cre*^; *Nlgn2*^*flox/flox*^ mice, *P* < 0.0001 for Control vs. *Lbx1*^*Cre*^; *Nlgn2*^*flox/+*^, *P* < 0.0001 for Control vs. *Lbx1*^*Cre*^; *Nlgn2*^*flox/flox*^, one-way ANOVAs with post hoc Dunnett’s tests. **b**, Response to a 0.9 PSI air puff stimulus alone. n = 18 control mice, n = 12 *Cdx2*^*Cre*^; *Nlgn2*^*flox/+*^ mice, n = 17 *Cdx2*^*Cre*^; *Nlgn2*^*flox/flox*^ mice, *P* = 0.0367 for Control vs. *Cdx2*^*Cre*^; *Nlgn2*^*flox/+*^, *P* = 0.0012 for Control vs. *Cdx2*^*Cre*^; *Nlgn2*^*flox/flox*^. n = 13 control mice, n = 14 *Lbx1*^*Cre*^; *Nlgn2*^*flox/+*^ mice, n = 14 *Lbx1*^*Cre*^; *Nlgn2*^*flox/flox*^ mice, *P* = 0.0181 for Control vs. *Cdx2*^*Cre*^; *Nlgn2*^*flox/+*^, *P* = 0.0383 for Control vs. *Cdx2*^*Cre*^; *Nlgn2*^*flox/flox*^, *P* = 0.0014 for Control vs. *Lbx1*^*Cre*^*; Nlgn2*^*flox/+*^, *P* = 0.0174 for Control vs. *Lbx1*^*Cre*^; *Nlgn2*^*flox/flox*^, Kruskal-Wallis tests with post hoc Dunn’s tests. **c**, Discrimination indices for textured NORT. n = 15 control mice and n = 11 *Cdx2*^*Cre*^; *Nlgn2*^*flox/flox*^ mice, *P* = 0.0357; n = 24 control mice and n = 12 *Lbx1*^*Cre*^; *Nlgn2*^*flox/flox*^ mice, *P* = 0.0028, unpaired two-tailed t-tests. **d**, Distance traveled in the open field. n = 15 control and n = 21 *Advillin*^*Cre*^; *Gabrb3*^*flox/flox*^ mice*, P* = 0.1222; n = 18 control and n = 13 *Lbx1*^*Cre*^; *Gabrb3*^*flox/flox*^ mice, *P* = 0.4756;; n = 15 control and n = 15 *Cdx2*^*Cre*^; *Nlgn2*^*flox/flox*^ mice, *P* = 0.2015; n = 22 control and n = 11 *Advillin*^*Cre*^; *Nlgn2*^*flox/flox*^ mice, *P* = 0.1862; n = 13 control and n = 13 *Lbx1*^*Cre*^; *Nlgn2*^*flox/flox*^ mice, *P* = 0.6495, unpaired two-tailed t-tests. **e**, Percent preference for a chamber containing a novel mouse versus a novel object in the 3-chamber social interaction assay. n = 14 control mice and n = 7 *Advillin*^*Cre*^; *Gabrb3*^*flox/flox*^ mice, *P* = 0.0217; n = 16 control mice and n = 12 *Lbx1*^*Cre*^; *Gabrb3*^*flox/flox*^ mice, *P* = 0.8816; n = 8 control mice and n = 11 *Cdx2*^*Cre*^; *Nlgn2*^*flox/flox*^ mice, *P* = 0.7776, unpaired two-tailed t-tests. **f**, Displacement responses to a single presentation of 0.10, 0.25, 0.50, 0.75 and 1.0 PSI stimuli for P4 *Advillin*^*Cre*^; *Gabrb3*^*flox/flox*^ (left) and *Cdx2*^*Cre*^; *Nlgn2*^*flox/flox*^ (right) and control littermate animals. Each dot represents the displacement response of a single animal on an air puff trial, and dashes represent means. n = 21 control and n = 19 *Advillin*^*Cre*^; *Gabrb3*^*flox/flox*^ mice; n = 17 control and n = 21 *Cdx2*^*Cre*^; *Nlgn2*^*flox/flox*^ mice. For **a**-**e**, data represent means ± s.e.m. Statistical information provided in Supplementary Table 1.

We next asked whether *Nlgn2* is required in spinal cord touch circuits for normal tactile responsiveness. We observed robust expression of NLGN2 in the spinal cord (Fig. 4a) and therefore generated *Cdx2*^Cre^; *Nlgn2*^fl/fl^^33,53^ mice to conditionally delete *Nlgn2* in all cells caudal to cervical level 2 of the spinal cord (Fig. 4a). Unlike *Avil*^Cre^; *Nlgn2*^fl/fl^ mice, *Cdx2*^Cre^; *Nlgn2*^fl/fl^ animals recapitulated the tactile behavioral deficits observed in the *Nlgn2* global knockout animals, thus revealing a novel locus of NLGN2 function outside of the brain in regulating tactile behaviors in mice (Fig. 4c,d and Extended Data Fig. 5a–c). We turned our attention to the possibility that *Nlgn2* is required in spinal cord dorsal horn neurons, where light touch information is processed, for tactile behaviors in adult animals. To drive selective loss of *Nlgn2* from this region, we used *Lbx1*^Cre^^54^, which promotes recombination in ~95% of all neurons in the LTMR-recipient zone (LTMR-RZ) of the dorsal horn^40^ (Fig. 4a). Loss of *Nlgn2* from dorsal horn neurons in *Lbx1*^Cre^; *Nlgn2*^fl/fl^ mice was sufficient to recapitulate the tactile behavioral alterations observed in both global *Nlgn2*-knockout and *Cdx2*^Cre^; *Nlgn2*^fl/fl^ animals (Fig. 4c,d and Extended Data Fig. 5a–c). By contrast, *Lbx1*^Cre^; *Gabrb3*^fl/fl^ animals, in which the β3 subunit of GABA_A_Rs is lost from dorsal horn neurons (Fig. 4b), showed normal reactivity to light touch stimuli (Fig. 4c,d). This may be consistent with a predominant role for glycinergic inhibition of light touch-evoked responses in the dorsal horn^55^ and/or the presence of GABA_A_Rs that do not include the β3 subunit that are important for touch behaviors.

In keeping with our observations in adult global *Nlgn2*-mutant animals, neither sensory nor spinal cord neuron loss of *Nlgn2* expression affected anxiety-like or social behaviors, despite tactile overreactivity in the spinal cord *Nlgn2* mutants (Fig. 4e and Extended Data Fig. 5d,e). By contrast, sensory neuron, but not spinal cord neuron, loss of *Gabrb3* expression promoted aberrant tactile, anxiety-like and social behaviors, consistent with a specialized role for this gene in peripheral sensory neurons (Fig. 4e and Extended Data Fig. 5d,e). Thus, although *Gabrb3* functions cell autonomously in peripheral somatosensory neurons, the ASD-associated gene *Nlgn2* functions in dorsal horn neurons, revealing a role for *Nlgn2* at an initial site of touch information processing in the central nervous system. Taken together with prior findings, these results indicate that both *Gabrb3* and *Mecp2* are required in peripheral somatosensory neurons^12,22,23^, while *Nlgn2*- and *Rorb*-expressing neurons are required in the dorsal horn for tactile behaviors in adults^39^.

### Locus of dysfunction predicts tactile overreactivity

To understand whether these distinct loci of function of the ASD-associated genes contribute to the timing differences for tactile overreactivity during development, we next tested the reactivity of conditional knockout animals to an air puff stimulus at P4. As with P4 *Gabrb3*^+/–^ animals, P4 *Avil*^Cre^; *Gabrb3*^fl/fl^ animals exhibited overreactivity to air puff stimuli, and these conditional mutant animals also had deficits in habituation to repeated stimuli (Fig. 4f–h, Extended Data Fig. 5f and Supplementary Video 1). Thus, the cell-autonomous requirement for *Gabrb3* in peripheral somatosensory neurons emerges early in development, and its disruption leads to developmental overreactivity to light tactile stimuli. By contrast, and consistent with our analyses of P4 *Nlgn2*^+/–^ animals, *Cdx2*^Cre^; *Nlgn2*^fl/fl^ P4 animals were indistinguishable from control littermates in their tactile reactivity (Fig. 4f–h and Extended Data Fig. 5f). Importantly, both *Avil*^Cre^- and *Cdx2*^Cre^-mediated recombination occur during embryonic development and are complete before birth^53,56^. Therefore, peripheral somatosensory neuron *Gabrb3* expression is required for normal tactile reactivity during early postnatal development, whereas spinal cord *Nlgn2* expression is dispensable during early postnatal development but is required in adulthood.

### Early GABA_A_R function in peripheral sensory neurons

Primary somatosensory neuron-specific deletion of *Gabrb3* was sufficient to drive behavioral overreactivity to light air puff stimuli in neonatal pups, indicating a developmental and cell-autonomous role for *Gabrb3* in peripheral sensory neurons. In adults, sensory neuron-derived GABRB3 localizes in the proximity of VGLUT1^+^ sensory neuron axon terminals in the spinal cord to form GABA_A_Rs, which underlie presynaptic inhibition of these neurons^22,23,55^. We asked whether we would observe a similar pattern of GABRB3 expression in the neonatal spinal cord. Using immunohistochemical analyses, we found that GABRB3 was detectable in the spinal cord at P0–P1 (Extended Data Fig. 2d) and was localized in the proximity of VGLUT1^+^ axon terminals (Extended Data Fig. 2e), and the relationship between GABRB3 and VGLUT1 at this time appeared similar to that observed in adult spinal cord tissue (Extended Data Fig. 2e,f). Furthermore, the proximity values between GABRB3 and VGAT^+^ presynaptic inhibitory interneuron axon terminals were similar between neonates and adults. These analyses were performed to compare the degree of proximity between synaptic markers across neonatal and adult time points, and the values reported in each condition likely overestimate true synaptic colocalization values due to the optical resolution limits of confocal microscopy. Nevertheless, these findings suggest that the synaptic machinery underlying presynaptic inhibition of sensory afferents is in place by birth or earlier (Extended Data Fig. 2f).

**Fig. 5:**
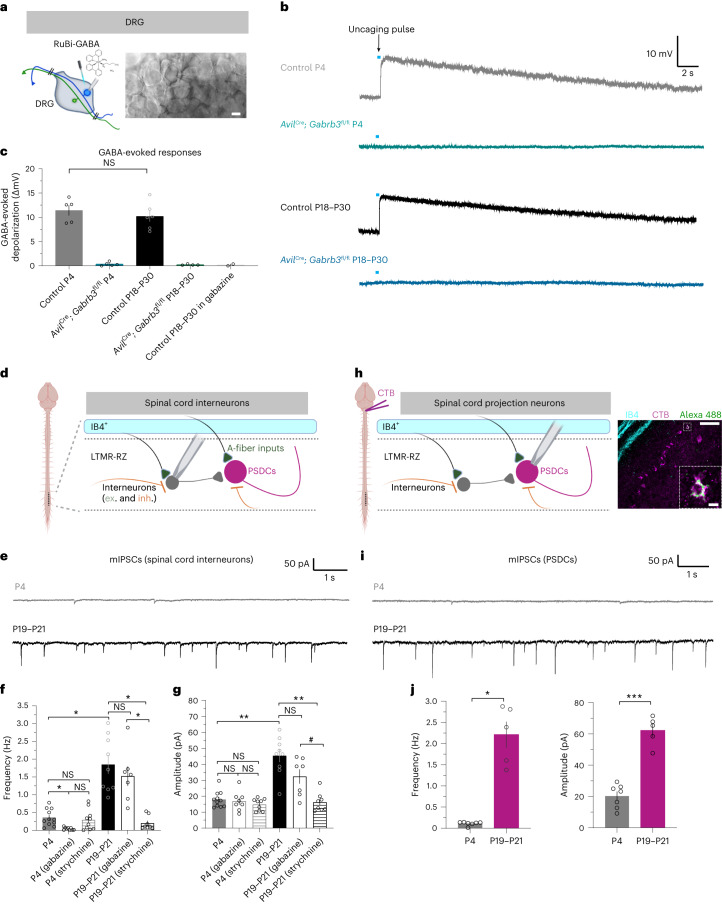
Machinery for presynaptic inhibition of sensory neurons develops neonatally, whereas spinal cord feedforward inhibition is weak and immature during early postnatal development. **a**, Experimental setup and example image of a DRG whole-cell recording preparation for RuBi-GABA uncaging; scale bar, 25 µm. **b**, Example GABA-evoked depolarizations of medium- to large-diameter neurons to a 5-ms uncaging pulse (blue bar, not to scale) in current clamp configuration. **c**, Quantification of peak uncaged GABA-evoked depolarizations across time points and genotypes. Each dot represents a cell, and *n* = 5 for control P4, *n* = 5 for *Avil*^Cre^; *Gabrb3*^fl/fl^ P4, *n* = 6 for control P18–P30, *n* = 4 for *Avil*^Cre^; *Gabrb3*^fl/fl^ P18–P30 and *n* = 2 control P18–P30 in gabazine. *N* = 2 animals for each condition, except *N* = 3 for control P18–P30. *P* = 0.4592, as determined by unpaired two-tailed *t*-test. **d**, Recording schematic for measuring miniature postsynaptic currents from interneurons (both excitatory and inhibitory, denoted ex. and inh., respectively) in the LTMR-RZ (laminae III–IV, which resides underneath the IB4^+^ superficial lamina II) of sagittal spinal cord slices. Parts created with BioRender.com. **e**, Example mIPSCs in P4 and P19–P21 spinal cords. Recordings were performed in laminae III/IV of sagittal spinal cord sections. **f**,**g**, mIPSC frequencies (**f**) and amplitudes (**g**) at P4 and P19–P21, with or without the presence of blockers of synaptic inhibition, gabazine and strychnine. Each dot represents a cell, and *n* = 11 for P4, *n* = 8 for P4 (gabazine), *n* = 9 for P4 (strychnine), *n* = 9 for P19–P21, *n* = 7 for P19–P21 (gabazine) and *n* = 8 for P19–P21 (strychnine). *N* = 3 animals for each condition, except *N* = 2 for P4 (gabazine), P4 (strychnine) and P19–P21 (strychnine). For frequencies, *P* = 0.0075 for P4 versus P4 (gabazine), *P* = 0.9959 for P4 versus P4 (strychnine), *P* = 0.1404 for P4 (gabazine) versus P4 (strychnine), *P* = 0.0024 for P4 versus P19–P21, *P* = 0.9601 for P19–P21 versus P19–P21 (gabazine), *P* = 0.0011 for P19–P21 versus P19–P21 (strychnine) and *P* = 0.0134 for P19–P21 (gabazine) versus P19–P21 (strychnine). For amplitudes, *P* = 0.9994 for P4 versus P4 (gabazine), *P* = 0.5713 for P4 versus P4 (strychnine), *P* = 0.9673 for P4 (gabazine) versus P4 (strychnine), *P* = 0.0005 for P4 versus P19–P21, *P* = 0.2662 for P19–P21 versus P19–P21 (gabazine), *P* = 0.0002 for P19–P21 versus P19–P21 (strychnine) and *P* = 0.0577 for P19–P21 (gabazine) versus P19–P21 (strychnine). Data were analyzed by Welch’s analysis of variance with Dunnett’s T3 multiple comparisons tests. **h**, Cholera toxin subunit B (CTB) was injected into the dorsal column or dorsal column nuclei to retrogradely label PSDCs. Sagittal spinal cord slices were prepared, and PSDCs were targeted for whole-cell patch clamp recordings (left) and, in some cases, dialyzed with Alexa Fluor 488 dye for post hoc visualization (right); scale bars, 100 µm (full-size image) and 10 µm (inset). Parts created with BioRender.com. **i**, Example mIPSCs from CTB-labeled PSDCs in P4 and P19–P21 spinal cords. **j**, mIPSC frequencies (left) and amplitudes (right) at P4 and P19–P21 onto PSDCs. Each dot represents a cell, and *n* = 7 for P4 and *n* = 5 for P19–P21. *N* = 3 animals for both conditions. For frequencies, *P* = 0.0024. Data were analyzed by unpaired two-tailed Welch’s *t*-test. For amplitudes, *P* < 0.0001. Data were analyzed by unpaired two-tailed *t*-test. For **c**, **f**, **g** and **j**, data are shown as mean ± s.e.m. Statistical information is provided in Supplementary Table 1; ^#^*P* < 0.1; *P < 0.05; **P < 0.001; ***P < 0.0001. Source data

**Extended Data Fig. 6:**
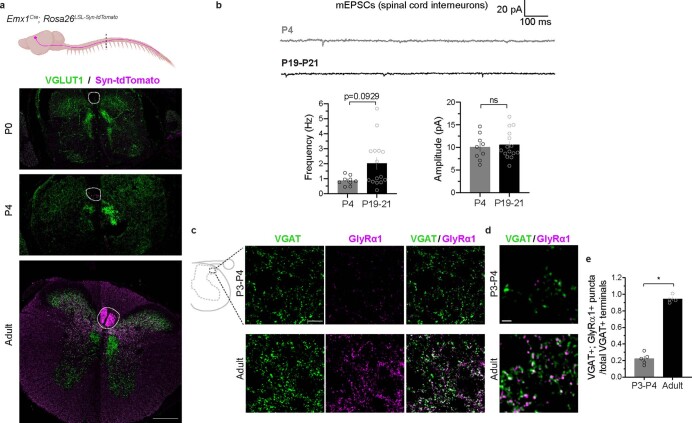
Synaptic elements for spinal cord inhibition emerge at different times, and additional characterization of neurotransmission in developing spinal cords. Related to Fig. 5. **a**, Anatomical labeling of the corticospinal tract in thoracic spinal cords of P0, P4, and adult animals labeled by in *Emx1*^*Cre*^; *Rosa26*^*LSL-synaptophysin-tdTomato*^. Scale bar denotes 200 µm. Parts created with BioRender.com. **b**, Example miniature excitatory post-synaptic currents (mEPSCs) from P4 and P19-21 spinal cord interneurons of lamina III/IV (top), and quantification of frequencies (bottom, left) and amplitudes (bottom, right). Each dot represents a cell, and n = 9 cells for P4 (N = 2 mice) and n = 15 cells for P19-P21 (N = 3 mice). For frequencies, *P* = 0.0929, two-tailed Mann-Whitney *U* test. For amplitudes, *P* = 0.6822, unpaired two-tailed t-test. **c**,**d**, Spinal cord immunohistochemistry showing expression of VGAT-labeled synaptic terminals and GLYRα1 in P3-P4 and adult mice. Scale bars denote 10 µm (**c**) and 2 µm (**d**). **e**, Quantification of VGAT and GLYRα1 co-labeled puncta, as a fraction of total VGAT+ puncta in a field of view, at P3-P4 or adulthood. Each dot represents the average value for one animal, with three spinal cord images taken per animal. N = 5 P3-P4 mice and N = 4 adult mice, *P* < 0.0001, unpaired two-tailed t-test. For **b** and **e**, data represent means ± s.e.m. Statistical information provided in Supplementary Table 1.

To complement the histological analyses, we also asked whether peripheral sensory neurons are functionally responsive to GABA application at early postnatal ages and, if so, whether these responses require *Gabrb3* expression. To test this, we performed whole-cell patch clamp recordings of DRG neurons using an ex vivo preparation and uncaged light-sensitive caged RuBi-GABA over the soma of recorded neurons (Fig. 5a). Medium- to large-diameter sensory neurons (>35 μm in diameter, >55 pF capacitance)^57^, which underlie low-threshold mechanosensory responses, were targeted and dialyzed with a high-chloride internal solution. Light pulses (5 ms) consistently drove large depolarizations in these sensory neurons at P4, and their magnitudes were not significantly different from P18–P30 neuron responses (Fig. 5b,c). Furthermore, at both time points, GABA-evoked responses in medium- and large-diameter DRG neurons of *Avil*^Cre^; *Gabrb3*^fl/fl^ animals were virtually abolished, highlighting the obligatory role of GABRB3 for the formation of functional GABA_A_Rs in these peripheral sensory neurons (Fig. 5b,c). Thus, GABA_A_Rs are present on medium- and large-diameter DRG neurons during neonatal development and require *Gabrb3* expression for responses to GABA. Interestingly, although presynaptic GABA_A_Rs are present early in development, top–down cortical inputs into the dorsal horn, which engage presynaptic inhibition of primary afferents and other aspects of local touch processing in adulthood^40,58^, were anatomically absent (Extended Data Fig. 6a). Together, our immunohistochemical and electrophysiological analyses indicate that the synaptic machinery underlying presynaptic inhibition of primary somatosensory neuron afferents matures by neonatal ages, supporting the idea that ASD-associated mutations impacting presynaptic inhibition disrupt sensory neuron function and tactile reactivity very early in development.

### Spinal cord feedforward inhibition is immature at early ages

In contrast to *Gabrb3*- and *Mecp2*-mutant animals, *Nlgn2*- and *Rorb*-mutant animals exhibited normal tactile behaviors during early postnatal development. However, spinal cord-specific disruptions of *Nlgn2* and *Rorb* neurons drive aberrant reactivity in adulthood^39^ (Fig. 4c,d). We therefore hypothesized that the spinal cord functions mediated by *Nlgn2* and *Rorb* develop after early postnatal periods, in contrast to the early developmental sensory neuron functions of *Gabrb3* and *Mecp2*^23^. Because both *Nlgn2* and *Rorb* are implicated in feedforward inhibitory neurotransmission^31,39,59^, we asked whether feedforward inhibition of dorsal horn LTMR-RZ interneurons and projection neurons (laminae III–IV of the spinal cord) was present at early neonatal stages. Therefore, we measured miniature inhibitory postsynaptic currents (mIPSCs) in laminae III–IV of neonatal (P4) and mature (P19–P21) animals (Fig. 5d). At P4, the frequency and amplitude of mIPSCs onto spinal cord neurons were dramatically lower than those of P19–P21 spinal cord neurons (Fig. 5e–g). Furthermore, although application of the GABA_A_R antagonist gabazine blocked nearly all mIPSCs at P4, gabazine did not strongly affect mIPSC frequency in recordings from P19–P21 mice (Fig. 5f). Application of the glycine receptor antagonist strychnine reduced both the frequency and amplitude of inhibitory events in mature slices, but not at P4 (Fig. 5f,g). By contrast, AMPA receptor-mediated miniature excitatory postsynaptic currents (mEPSCs) in spinal cord neurons were more similar early and late in development (Extended Data Fig. 6b).

In complementary experiments, we recorded mIPSCs from retrogradely labeled postsynaptic dorsal column neurons (PSDCs), which are dorsal horn output neurons that project to the brainstem^60^ and receive direct inputs from Aβ-LTMRs, other LTMR subtypes and spinal cord interneurons^39,40^ (Fig. 5h). Consistent with randomly recorded laminae III–IV neurons, the frequency and amplitude of mIPSCs onto PSDCs from P4 pups were markedly lower than those onto mature PSDCs from P19–P21 mice (Fig. 5i,j).

Finally, measurements of VGAT^+^ inhibitory interneuron axon terminals and postsynaptic α1 subunit-containing glycine receptors in the spinal cord revealed that immunohistochemical proximity between VGAT and glycine receptors was significantly lower at P4 than that in adulthood^55^ (Extended Data Fig. 6c–e). Together, these findings indicate that feedforward inhibition in the LTMR-RZ develops late; inhibition is weak and mediated solely by GABA at P4, and it greatly strengthens and becomes predominantly glycinergic by P19. These findings are consistent with prior analyses performed in the superficial dorsal horn of early neonatal rat spinal cords, where nociceptive inputs impinge on spinal neurons free of glycinergic inhibition, which matures over the second postnatal week^61^.

### *Nlgn2* is required for spinal cord feedforward inhibition

**Fig. 6:**
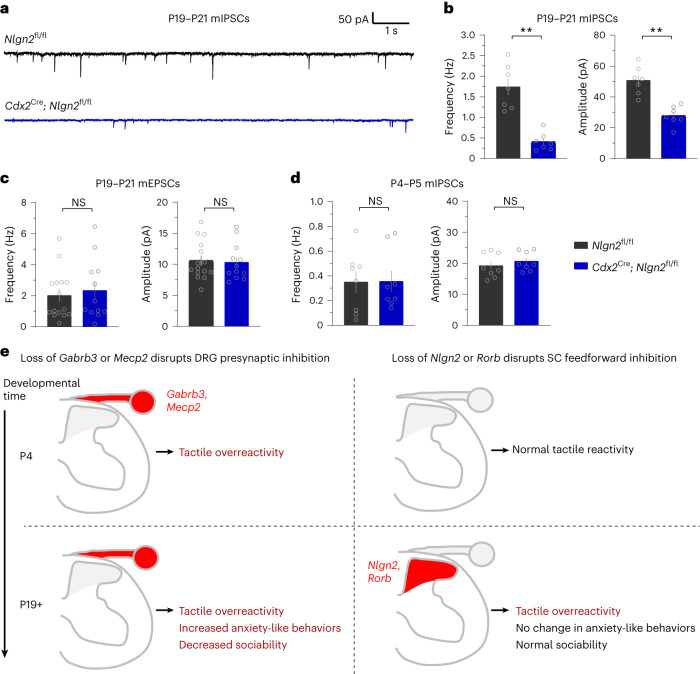
NLGN2 is required for feedforward inhibitory neurotransmission in the mature spinal cord dorsal horn. **a**, Example mIPSCs in spinal cord neurons of P19–P21 spinal cords in *Cdx2*^Cre^; *Nlgn2*^fl/fl^ animals and control littermates. **b**, mIPSC frequencies (left) and amplitudes (right) of P19–P21 spinal cords in *Cdx2*^Cre^; *Nlgn2*^fl/fl^ animals and control littermates. Each dot represents a cell, and *n* = 7 *Nlgn2*^fl/fl^ cells and *n* = 7 *Cdx2*^Cre^; *Nlgn2*^fl/fl^ cells. *N* = 2 animals for both conditions. For frequencies, *P* = 0.0003. Data were analyzed by unpaired two-tailed Welch’s *t-*test. For amplitudes, *P* = 0.0001. Data were analyzed by unpaired two-tailed *t*-test. **c**, Frequencies (left) and amplitudes (right) of mEPSCs in the spinal cords of *Cdx2*^Cre^; *Nlgn2*^fl/fl^ animals and control littermates at P19–P21. Each dot represents a cell, and *n* = 15 *Nlgn2*^fl/fl^ cells and *n* = 12 *Cdx2*^Cre^; *Nlgn2*^fl/fl^ cells. *N* = 2 animals for both conditions. For frequencies, *P* = 0.6052. Data were analyzed by two-tailed Mann–Whitney *U*-test. For amplitudes, *P* = 0.6822. Data were analyzed by unpaired two-tailed *t*-test. **d**, Frequencies (left) and amplitudes (right) of mIPSCs in the spinal cords of *Cdx2*^Cre^; *Nlgn2*^fl/fl^ animals and control littermates at P4–P5. Each dot represents a cell, and *n* = 8 *Nlgn2*^fl/fl^ cells and *n* = 8 *Cdx2*^Cre^; *Nlgn2*^fl/fl^ cells. *N* = 2 animals for both conditions. *P* = 0.9678 for frequencies, and *P* = 0.4385 for amplitudes. Data were analyzed by unpaired two-tailed *t*-tests. **e**, Proposed model describing the emergence of DRG presynaptic inhibition and spinal cord (SC) feedforward inhibition across development, with observed behavioral consequences of disrupting ASD-associated genes involved in either pathway. For **b**–**d**, data are shown as mean ± s.e.m. Statistical information is provided in Supplementary Table 1; **P < 0.001. Source data

**Extended Data Fig. 7:**
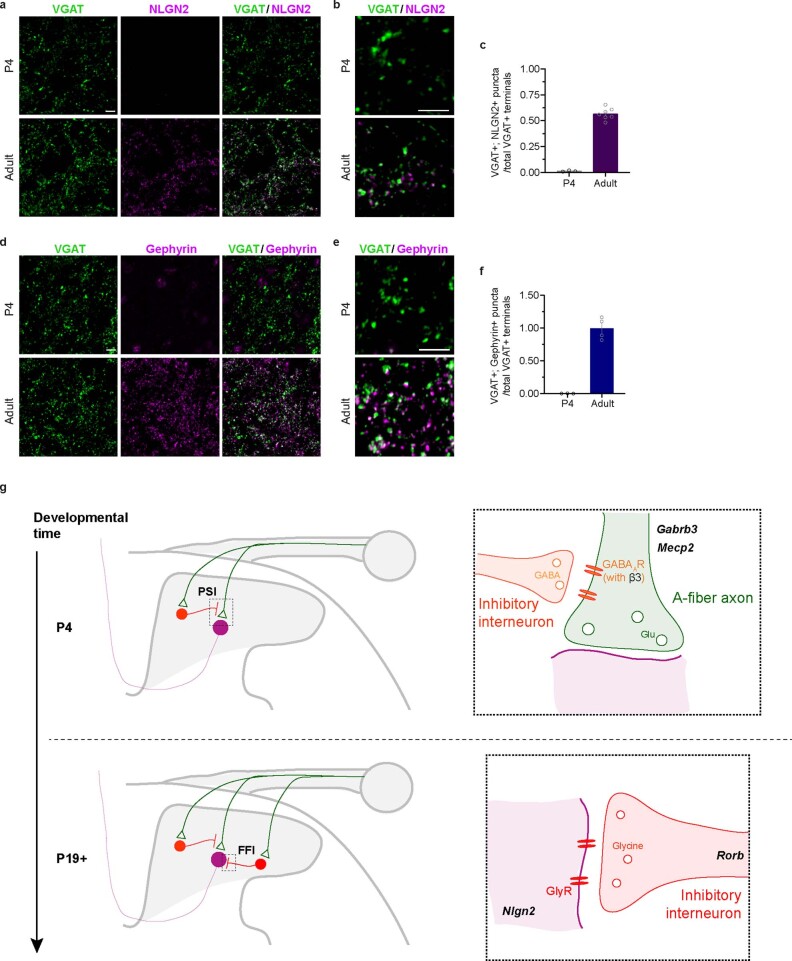
NLGN2 and gephyrin immunoreactivity are lacking at P4. Related to Fig. 6. **a**, **b**, Spinal cord immunohistochemistry of VGAT and NLGN2 in P4 and adult animals. Scale bars denote 5 µm. **c**, Quantification of VGAT/NLGN2 co-labeling. **d**, **e**, Spinal cord immunohistochemistry of VGAT and gephyrin in P4 and adult animals. Scale bars denote 5 µm. **f**, Quantification of VGAT/gephyrin co-labeling. For **c** and **f**, Each dot represents the average value for one animal, with three spinal cord images taken per animal. N = 3 P3-P4 mice and N = 7 adult mice for NLGN2, and N = 3 P3-P4 mice and N = 4 adult mice for gephyrin. Data represent means ± s.e.m. **g**, Proposed model for the molecular and circuit development of presynaptic inhibition (PSI) and feedforward inhibition (FFI) in the spinal cord. Glu denotes glutamate, and GlyR denotes glycine receptor.

Our behavioral findings demonstrate that NLGN2 is required for normal tactile reactivity in adulthood but not at P4, and our electrophysiological recordings indicate that feedforward inhibitory synaptic transmission in the LTMR-RZ is robust in the adult dorsal horn but is nearly absent at P4. Thus, we hypothesized that NLGN2 functions during late postnatal development of the dorsal horn LTMR-RZ to control the maturation of inhibitory transmission. In spinal cord slice preparations, mIPSC recordings in the LTMR-RZ revealed that both the frequency and amplitude of mIPSCs were markedly reduced in P19–P21 *Cdx2*^Cre^; *Nlgn2*^fl/fl^ slices compared to controls (Fig. 6a,b). Consistent with previous work in other central nervous system regions^30,33,62–64^, this change was specific to inhibitory neurotransmission; mEPSC frequency and amplitude were normal in the same preparation (Fig. 6c). Thus, in late postnatal mice, *Nlgn2* is required for feedforward inhibitory neurotransmission in the spinal cord dorsal horn, where light touch signals are first relayed. By contrast, and consistent with our behavioral findings, recordings in P4–P5 *Cdx2*^Cre^; *Nlgn2*^fl/fl^ and littermate control spinal cords revealed no significant differences in mIPSC frequency or amplitude between groups (Fig. 6d). Finally, immunohistochemical analyses of the expression of both NLGN2 and gephyrin, an inhibitory postsynaptic scaffolding protein, in the neonatal spinal cord showed no detectable signal at P4, despite robust expression in adulthood (Extended Data Fig. 7a–f). These electrophysiological and histological analyses indicate that NLGN2 is lowly or not expressed and is dispensable for the weak and immature feedforward inhibition present in the early postnatal deep dorsal horn, consistent with the observation that *Nlgn2* deletion does not affect tactile reactivity during early postnatal development. By contrast, by late postnatal ages and adulthood, NLGN2 is robustly expressed and required for normal feedforward inhibitory transmission in the spinal cord, which is primarily glycinergic. Thus, the cellular and synaptic mechanisms underlying glycinergic feedforward inhibition and GABAergic presynaptic inhibition in the spinal cord mature at different stages.

These findings support a model in which ASD-associated gene mutations affecting presynaptic inhibition of LTMRs manifest during late embryonic and early neonatal development and thereby have profound effects on embryonic and early postnatal reactivity to tactile stimuli. By contrast, ASD-associated gene mutations affecting dorsal horn feedforward inhibition manifest later, during late postnatal development, and thus are without deleterious consequence on early postnatal tactile reactivity (Fig. 6e and Extended Data Fig. 7g). Importantly, animals with neonatal tactile overreactivity also exhibit increased anxiety-like behaviors and decreased sociability in adulthood, whereas animals with normal early tactile reactivity do not exhibit these changes.

## Discussion

Here, we report that distinct cellular and synaptic mechanisms in peripheral somatosensory neurons and spinal cord neurons contribute to tactile overreactivity in mouse models of ASDs. Mice with deletion of the ASD-associated genes *Gabrb3* and *Mecp2*, which are required for presynaptic inhibition onto peripheral somatosensory afferents^12,22,23^, exhibit aberrant tactile reactivity from early neonatal stages. By contrast, disruptions in *Nlgn2* or *Rorb*, which impact feedforward inhibition of spinal cord dorsal horn neurons^39^, do not affect tactile reactivity neonatally. Although all of these animal models exhibit aberrant touch behaviors in adulthood, mice that had tactile overreactivity in early life also present with anxiety-like behaviors and sociability deficits in adulthood, whereas mice with normal tactile reactivity in early life do not exhibit these additional alterations. Therefore, in these models of ASD, developmental somatosensory dysfunction is linked to the emergence of cognitive and social behavior disruptions.

We found that the machinery for GABAergic presynaptic inhibition of large-diameter peripheral somatosensory neurons is present by early neonatal stages. Therefore, ASD-associated mutations that disrupt presynaptic GABA_A_ receptors impact somatosensory behaviors during development. To assess this, we developed an approach to quantitatively measure tactile reactivity in neonatal animals and found that the *Gabrb3* and *Mecp2* loss-of-function models exhibited tactile overreactivity to a gentle air puff stimulus at P4. This overreactivity can be attributed to a cell-autonomous role for GABAergic signaling in peripheral somatosensory neurons. Somatosensory neuron-specific deletion of *Gabrb3* ablated functional responsivity to GABA in sensory neurons, and mutant animals recapitulated the aberrant behaviors we observed in *Gabrb3*^+/–^ mice. Remarkably, *Gabrb3*^+/–^ mice also exhibited overreactivity to activation of Ret^+^ Aβ-LTMRs during late embryonic development, and so loss of primary afferent presynaptic inhibition may drive sensory overreactivity in utero. Consistent with this, a prior study detected GABAergic dorsal root potentials, a hallmark of presynaptic inhibition, as early as E17.5 in isolated rat spinal cords^65^. We therefore speculate that aberrant activity in tactile circuits in utero or during early postnatal development may contribute to the emergence of core and comorbid ASD features in certain individuals. This finding extends our prior work defining a critical period that ends during early postnatal life in mice, in which physiological dysfunction of primary sensory neurons impacts cortical microcircuits and drives enhanced anxiety-like behaviors and abnormal social behaviors^12^. In this prior work, embryonic or P5 deletion of *Mecp2* or *Shank3* led to alterations in tactile reactivity, anxiety-like behaviors and sociability in adulthood. By contrast, although deletion of these genes in sensory neurons beginning at P28 led to an increase in tactile reactivity in adulthood, this alteration was not accompanied by abnormal anxiety-like behaviors and sociability.

Although the machinery that underlies presynaptic inhibition of peripheral sensory neurons matures early in development, feedforward inhibitory neurotransmission onto central neurons in the spinal cord dorsal horn, where light touch is processed, matures across a much slower timescale. Our work in the mouse LTMR-RZ agrees with studies performed in the rat superficial spinal cord, showing that feedforward inhibition onto spinal cord neurons shifts from early GABAergic inputs to both glycinergic and GABAergic control over the course of postnatal development^61,66^. In the adult rat, LTMR-evoked responses are under strong glycinergic control^55,67^, and it is thought that both glycinergic inputs and postsynaptic receptors only reach maturity at the end of the second postnatal week^55,68^. Here, we identified *Nlgn2* as an important component in feedforward inhibition of dorsal horn neurons. Developmental deletion of *Nlgn2* did not impact spinal synaptic function or behavioral reactivity to low-threshold stimuli during early postnatal development, and NLGN2, the inhibitory postsynaptic scaffolding protein gephyrin and glycine receptor expression were nearly undetectable or at very low levels in the early postnatal spinal cord. The aberrant tactile behaviors observed in adult *Nlgn2* mutants likely corresponds to the late impact on the maturation of feedforward inhibitory circuitry, and future experiments that address whether the emergence of aberrant tactile behaviors precisely coincides with the onset of dorsal horn feedforward inhibition are warranted. We also investigated the ASD-associated gene *Rorb* because silencing *Rorb*-expressing spinal neurons leads to a dramatic loss of glycinergic feedforward inhibition onto PSDCs and promotes tactile dysfunction^39^. Similar to *Nlgn2*-mutant animals, *Rorb*-mutant animals had normal touch reactivity at P4 but exhibited enhanced tactile reactivity in adulthood. There was substantial correspondence in behavioral phenotypes between animals with *Rorb* gene mutations and silenced or ablated *Rorb* neurons^39^, but future studies should address whether spinal cord feedforward synaptic inhibition is impacted to the same extent under these two conditions. Furthermore, although deficits in spontaneous postsynaptic currents were observed in *Nlgn2*-mutant animals in the present study, it remains to be determined whether touch-evoked spiking activity is altered in the spinal cords of these *Nlgn2* mutants during postnatal development or adulthood^69^.

Our findings support a model in which NLGN2 is an essential organizer of glycinergic synapses^30^ that lie postsynaptic to inhibitory glycinergic *Rorb*-expressing neurons and other inhibitory interneurons of the deep dorsal horn (Extended Data Fig. 7g). Thus, *Nlgn2* and *Rorb* may function within a common feedforward inhibition pathway in the mature dorsal horn that regulates behavioral responses to touch after a critical time window of perinatal development, during which tactile signals shape the central circuits governing non-tactile ASD comorbidities^12^. Indeed, *Nlgn2* and *Rorb* heterozygous mutant animals exhibit normal anxiety-like and social behaviors in adulthood, although it is possible that these animals may exhibit ASD-associated behavioral alterations not examined here or in prior studies^43,70^. Together, we propose that the differences in the developmental timing of function of ASD-associated genes that act within the same circuitry, for example, *Gabrb3*, *Mecp2*, *Nlgn2* and *Rorb* in the DRG sensory neuron/dorsal horn circuitry, help to explain the heterogeneity of behavioral outcomes observed across ASD mouse models.

In humans with ASD, behavioral studies indicate that altered sensory behaviors often predict the severity of higher-order ASD or ASD-related traits^8,14,71–75^. Importantly, altered sensory reactivity can be detected during early development in subsets of children with ASD^76^, raising the possibility that sensory processing changes may represent an early clinically relevant marker for the emergence of future ASD-related traits in subgroups of individuals. Indeed, the concept of ‘chronogeneity’ describes the diverse clinical trajectories that individuals with ASD can take through development and onward, and posits that temporal and cross-sectional heterogeneity across individuals may represent a form of ‘informative variance’ that describes a more dynamic and well-powered understanding of ASDs^77^. Our findings lead us to propose that variability in spinal sensory circuit alterations in ASD, and therefore variable developmental timing of aberrant sensory behaviors, contributes to observed chronogeneity across individuals. Therefore, a richer understanding of how atypical sensory processing behaviors during early development relate to other core and comorbid features of ASDs may inform future diagnostic, prognostic and interventional strategies.

## Methods

### Experimental model and subject details

All procedures performed in this study were approved by the Harvard Medical School Institutional Animal Care and Use Committee and were performed in compliance with the Guide for Animal Care and Use of Laboratory Animals. Male and female mice of mixed genetic backgrounds (C57BL/6J, 129/SvEv and CD1) were used for these studies. Housing and testing conditions were maintained at ~21–22.8 °C with 40–60% humidity. For adult behavioral testing, mice were weaned and ear notched for genotyping at P21 (±2 days), and testing was performed beginning at 6 weeks of age and completed by 8 weeks of age. For embryonic and neonatal behavior, mice were toe clipped following testing and genotyped. For electrophysiology experiments, mice were toe clipped and genotyped before P4. Mice were group housed with littermates in standard housing on a 12-h light/12-h dark cycle.

### Mouse lines and genotyping

*Gabrb3* floxed mice were obtained from the Jackson Laboratory (008310) and were previously described^78^. *Gabrb3*-null mice were generated by crossing *Gabrb3* floxed mice with *E2a*^Cre^ mice from the Jackson Laboratory (003724). *Mecp2*-null mice were obtained from the Jackson Laboratory (003890) and were previously described^79^. *Nlgn2* floxed mice were obtained from the Jackson Laboratory (025544) and were previously described^33^. *Nlgn2*-null mice were generated by crossing *Nlgn2* floxed mice with *E2a*^Cre^ mice and were subsequently backcrossed to C57BL/6J mice for at least six generations. *Rorb*^h1/h1^ mice were obtained from the Jackson Laboratory (006948) and were previously described^42^. *Nlgn4*-null mice were obtained from the Mutant Mouse Resource & Research Centers Repository (BayGenomics, 010566-UCD) and were previously described^34^. *Avil*^Cre^ mice were obtained from F. Wang (Duke University) and were previously described^56^. *Cdx2*^Cre^ (009350)^53^, *E2a*^Cre^ (003724)^80^ and *Emx1*^*Cre*^ (005628)^81^ mice were obtained from the Jackson Laboratory and were previously described. *Lbx1*^Cre^ (MGI, 104867)^54^, *Ret*^*creER*^ (MGI, 97902)^49^, *TrkB*^*creER*^ (MGI, 997384)^51^ and *TrkC*^*creER*^ (MGI, 97385)^50^ animals were previously described. *Rosa26*^*LSL-synaptophysin-tdTomato*^ (Ai34; 012570) and *Rosa26*^*LSL-FSF-ReaChR-mCitrine*^ (024846) mice were obtained from Jackson Laboratory. CD1 animals were obtained from Charles River (022), and C57BL/6J animals were obtained from the Jackson Laboratory (000664). *Avil*^*FlpO*^ mice were previously described^48^. Proper expression of each floxed allele using each *cre* transgene was assessed using PCR. *Avil*^Cre^ and *Lbx1*^Cre^ animals with post-Cre excision expression in tail biopsy tissue or *Cdx2*^Cre^ animals with excision in ear punch tissue were excluded from analyses. Information on primers used in this study can be found in Supplementary Table 2.

### Tamoxifen treatment

Tamoxifen (Sigma, T5648) was dissolved in 100% ethanol to 20 mg ml^–1^ (30 min of vortexing at room temperature (RT)), mixed with an equal volume of sunflower seed oil (Sigma, S5007) and vacuum centrifuged for 30 min to evaporate the ethanol. When tamoxifen was delivered embryonically, progesterone (Sigma, P0130) was also added to the ethanol at a concentration of 10 mg ml^–1^. Tamoxifen (20 mg ml^–1^, with 10 mg ml^–1^ progesterone) aliquots in sunflower seed oil were stored at –80 °C until the day of use, when they were thawed 15 min at RT while protected from light. Pregnant mice received 3 mg of tamoxifen by oral gavage at E11.5 (to label Aβ-LTMRs using *Ret*^*creER*^), E12.5 (to label slowly adapting Aβ-LTMRs using *TrkC*^*creER*^) or E13.5 (to label rapidly adapting LTMRs and Aδ-LTMRs using *TrkB*^*creER*^).

### Behavioral testing

Male and female mice of mixed genetic backgrounds (C57BL/6J, 129/SvEv and CD1) were used for behavioral testing. The only exceptions were *Nlgn2* and *Mecp2* germline mutant mice, which were backcrossed for at least five generations to a C57BL/6J background. For adult behavioral testing, littermates from the same genetic crosses were used as controls for each group (therefore, a combination of Cre-positive, floxed-negative and Cre-negative, floxed-positive mice were used for conditional knockout animal experiments). Before adult behavior testing, mice were weaned and ear notched (Kent Scientific, INS750075-5) for genotyping at P21 (±2 days), and testing was performed beginning at 6 weeks of age and completed by 8 weeks of age. For embryonic and neonatal behavior, mice were toe clipped following testing and genotyped. For all behavioral assays and analyses, experiments were performed by investigators blinded to genotype. Mice were group housed with littermates in standard housing on a 12-h light/12-h dark cycle with free access to food and water. *cre* alleles were always kept on the paternal genome, and all animals from each genetic cross had mothers of the same genotype, with the exception of crosses where the mother harbored either one or two floxed alleles (for example, *Gabrb3*^fl/+^ or *Gabrb3*^fl/fl^ mothers). No behavioral differences were observed between any wild-type, single or double flox or Cre^+^ control groups. No differences between male and female mice of the same genotype were observed. Adult animals hemizygous for *Mecp2* were only compared to male control littermates. For adult behavioral testing, cages were changed once per week by an investigator and at least 3 days before the next behavioral assay. Before testing, animals were brought into the procedure room in their home cages and allowed to habituate for 30 min, and testing rooms were illuminated at ~30 lux. All testing materials were cleaned thoroughly with 70% ethanol before and between trials. Behavioral video footage was recorded using an overhead camera (Imaging Source DMK 22AUC03) and IC Capture 2.5.

### Open field test

Mice were placed into the center of a 40 cm × 40 cm × 40 cm matte black acrylic testing chamber with a matte white acrylic floor. Behavior was recorded from above for a duration of 10 min, and videos were recorded using custom MATLAB scripts to analyze the distance traveled and time spent in the center of the arena. Time spent in the center was measured as the percentage of time spent at a distance of >5 cm from the edges of the chamber.

### Novel object recognition test

The novel object recognition test (NORT) was performed as previously described^23^. For 2 days, mice were individually habituated to the acrylic testing chamber used for the open field assay for 10 min each day under dim lighting. The next day, a texture NORT was used as a measure of texture discrimination. During an initial learning phase, the mouse was placed into the testing chamber with two identical objects, either both ‘rough’ or ‘smooth’ plexiglass cubes (4 cm^3^) and visually identical, and allowed to freely explore the objects and chamber for 10 min. The mouse was then returned to its home cage for a 5-min retention period, during which the chamber and objects were cleaned with 70% ethanol, and one object was replaced with an object of novel texture (rough or smooth). The mouse was then placed back in the chamber for the 10-min test phase. *Nlgn2*-null mice were subjected to a color/shape NORT, in which the objects were wooden blocks that differed in shape and color.

Video recordings were acquired from above during both the learning and test phases. Custom MATLAB scripts tracked mouse position in the chamber, and time spent investigating objects in both phases was calculated. The preference for the novel object was calculated by measuring the time spent investigating the novel object divided by the time spent investigating both objects during the test phase. Mice that did not investigate both objects during the learning phase were excluded from analyses. Mice were whisker plucked 3 days before the start of habituation, which does not affect exploratory behaviors or total time spent investigating objects during the NORT^23^.

### PPI of the startle reflex assay

PPI was performed using the San Diego Instruments startle reflex system (SR-LAB Startle Response System) as described previously^23^. Mice were placed in one of two types of cylindrical enclosures just wide enough for the animal to turn around comfortably, either 3.8 cm or 2.8 cm in diameter. Enclosures were then placed within a soundproof chamber. A prepulse was delivered before an acoustic startle pulse (125 dB, 20 ms), and the mouse’s startle response was measured using an accelerometer mounted to the enclosure. For tactile PPI, the prepulse was a 0.9-psi air puff (50 ms) delivered at variable interstimulus intervals (50, 100, 250, 500 and 1000 ms) before the acoustic startle pulse. Following an acclimation phase (5 min), testing sessions were arranged in blocks consisting of (1) responses to acoustic pulse trials alone, (2) prepulse stimuli alone, and (3) pseudorandomized trials of prepulse/pulse (startle response), pulse alone (used for the percent PPI calculation) and no stimulation (to capture baseline movement). Intertrial intervals varied from 10 to 30 s. Maximal accelerometer responses (in millivolts) were recorded in the 100-ms window following the end of each stimulus, and percent PPI was calculated as %PPI = [1 – (startle response/pulse alone response)] × 100. The response to air puff alone was measured as (prepulse alone/pulse alone response) – (no stimulation/pulse alone response). Animals whose baseline movement was greater than 25% of their acoustic startle response were not included in the analyses (that is, these animals exhibited insufficient acoustic startle amplitudes). All percentage PPI data reported in this study are from the 250-ms interstimulus interval trials.

### Elevated plus maze

The elevated plus maze consisted of four arms, each 30 cm long × 5 cm wide with white acrylic floors and with two of the opposing arms with black acrylic walls that were 15 cm high. The maze stood on 40-cm-tall legs. Behavior video was recorded from above. The animal was placed at the center of the maze and was allowed to explore for 10 min. The amount of time spent in each arm was tracked using a custom MATLAB script. The fraction of time spent exploring the open arms was calculated as time spent in open arms/(time spent in open + closed arms).

### Three-chamber social interaction test

Sociability was assessed using the three-chamber social interaction assay. Mice were first allowed to explore a three-chambered acrylic box with openings between the chambers for 5 min (each compartment was 20 cm wide × 40 cm long × 22 cm high). The outside walls of the chamber were black and opaque, and the two inner dividers were clear acrylic. After habituation, the test mouse was moved to the center chamber, and clear partitions were put into place to block the entrances to the other two chambers. A novel mouse was then placed in a wire mesh cup in one chamber, while an identical empty wire mesh cup (‘object’) was placed on the other side. The partitions were then lifted, and the test mouse was free to explore for 10 min while video was recorded from above. The chambers containing the novel mouse and object were switched for each session. The time spent in each of the three chambers during each test session was tracked using custom MATLAB scripts. Preference for the novel mouse was calculated as the time spent investigating the novel mouse/(time spent investigating the novel mouse + object).

### Neonatal tactile sensitivity assay

All animals were group housed, with control and mutant animals in the same litters and cages. We did not observe any sex-related differences in P4 tactile reactivity in any genetic cross, so male and female mice of the same genotype were grouped together for the final analyses. Male P4 animals hemizygous for *Mecp2* (*Mecp2*^–/y^) were compared to both male and female P4 control littermates.

For behavioral testing, P4 animals were removed from their home cage, brought into the behavioral testing room and returned to the home cage and litters immediately after testing. Animals were placed on warm, lightweight wool batting fabric, and the back of the mouse was positioned 2–3 mm below affixed air tubing. A camera (FLIR Integrated Imaging Solutions, FL3-U3-13E4M-C) was mounted overhead, and videos were acquired at 120 frames per s using Point Grey FlyCap2. Air puff stimuli of increasing intensity (0.10, 0.25, 0.50, 0.75 and 1.0 psi, 50 ms each) were delivered sequentially with pseudorandomized interstimulus intervals lasting between 20 and 30 s using software and equipment from San Diego Instruments (SR-LAB Startle Response System). The 1.0-psi stimulus was presented ten times consecutively to test for average responsivity to the same stimulus and for habituation, totaling 14 trials (<7 min of testing) per animal. At the beginning of each session, an image of a ruler was used to ensure identical viewing distances across sessions. Videos were analyzed for peak body displacement 500 ms following the air puff stimulus using the Face-Rhythm analysis package (https://github.com/RichieHakim/face-rhythm). Displacement fields for optic flow analysis consisted of a large number of points for markerless positional tracking and were applied to the dorsal rear of mice. Peak displacements are reported in pixels. Trials in which the animal exhibited movement during the 10-frame baseline collection period before the stimulus were discarded during analysis. For habituation analyses, only animals that exhibited >20% of baseline movement to the first presentation of the 1.0-psi stimulus were included for analysis. To calculate whether an animal exhibited habituation, the average displacement from the first three 1.0-psi trials and average of the last three trials were compared, and animals exhibiting a >25% reduction in responsivity were considered to have habituated. The 25% cutoff was chosen based on data collected from control animals, and analyses using 20, 30 and 40% cutoffs yielded similar trends in results. The experimenter was blind to the genotype of all test animals, as animals were toe clipped and genotyped after testing was completed.

For experiments related to Extended Data Fig. 2, 4% topical lidocaine (Henry Schein, 7280001) was applied to the back hairy skin of experimental animals following pretreatment data collection, and the animal was returned to its home cage for 1 h before testing. For hair removal experiments, the back hairy skin was treated with commercial depilatory cream (NAIR, Church and Dwight) for 1 min, and skin was gently cleaned with a dampened Kimwipe. The animal was then returned to its home cage for 1 h before testing.

### Embryonic and neonatal optical reactivity assay

To measure optically driven responses in ReaChR-labeled animals, a C-section was performed at E18.5, and pups were allowed to recover on a warm pad for 15–30 min before behavioral testing. Animals were placed with their paws below them and slightly resting on one side on a warmed clear sheet of acrylic. A single 50-ms pulse of 470-nm light (1.1 mW mm^–2^; Thor Labs, M470F3) through a Ø400-µm Core, 0.39-NA patch cable (Thor Labs, M79L01) was used to deliver stimuli five times with 20- to 30-s interstimulus intervals. We report peak displacements in the 500 ms following stimulus onset, and average displacements were calculated as the average peak displacement to five optical stimuli. Only the left or right paw was used for each animal. If an animal fell over in the 500 ms following the stimulus onset, then a maximal displacement value of 106.383 pixels was used, which reflects a maximum displacement tracked before the tracking points become occluded. In the case of an animal falling over, the animal would be gently righted during the interstimulus interval. Following the experiments, animals were cross-fostered to a CD1 female with an age-matched litter, and some animals were tested again at P0. Most P0 testing occurred in a separate set of animals that were delivered naturally or via C-section. No differences were observed between animals of the same genotype that were tested at both E18.5 and P0 versus only P0. Animals were toe clipped and genotyped after testing was completed.

### Immunohistochemistry

For histology in wild-type animals, C57BL/6J mice were used for each experiment. For mutant versus control analyses, littermates were killed in pairs or groups. Both males and females were used. For adult experiments, mice (P42–P84) were weighed, anesthetized with isoflurane and transcardially perfused with 0.25 ml per gram of body weight Ames Media (Sigma, A1420) in PBS with heparin (10 U ml^–1^; Sigma, H3393-100KU), followed by 0.5 ml per gram of body weight 2% paraformaldehyde (PFA; Millipore Sigma, P6148) in PBS at RT. No postfixation was performed, and spinal cords and/or DRG were finely dissected from perfused mice.

For P0 and P4 spinal cord analyses in C57BL/6J animals, pups were deeply anesthetized in an ice bath for several minutes before killing. Mice were decapitated, and the spinal column was dissected and drop-fixed in 2% PFA for 1 h at RT. The tissue was then washed several times over 1 h in PBS. All spinal cord analyses were performed on lumbar segments (L3–L6).

Both adult and neonatal tissue was cryoprotected in 30% sucrose in PBS for two nights at 4 °C, embedded in NEG-50 (VWR, 84000-156) and frozen in tissue molds over a dry ice and ethanol slurry. Tissue was stored at –80 °C until transverse sections at 25 µm were acquired using a cryostat, with littermate controls and mutants on the same slides or C57BL/6J animals of different ages (P0, P4 and adult) on the same slides. Slides were allowed to dry overnight at RT before staining or were stored at –20 °C until staining.

After overnight drying or defrosting frozen samples for 15 min at RT, slides were rehydrated with three 5-min washes in PBS. For tdTomato and MECP2 staining, slides were blocked for 2 h in PBS containing 0.1% Triton X-100 and 5% normal goat or donkey serum (Jackson ImmunoResearch, 005-000-121 and 017-000-121, respectively). Sections were incubated with primary antibodies diluted in blocking solution (5% goat or donkey serum with no detergent) overnight at 4 °C. Slides were washed four times for 5 min each in PBS containing 0.02% Tween 20 and then incubated with species-specific secondary antibodies in blocking solution for 1 h at RT. The tissue was washed with PBS containing 0.02% Tween 20 four times for 5 min each, mounted and coverslipped with Fluoromount Aqueous Mounting Medium with DAPI (Thermo Fisher, 00-4959-52). Slides were stored at 4 °C.

For GABRB3, glycine receptor subunit α1, NLGN2 and gephyrin stains, a high-salt protocol with antigen retrieval steps was used. High-salt PBS (HS-PBS) was composed of 0.3 M NaCl PBS. Tissue sections were incubated in 50% ethanol in double distilled water for 30 min to improve antibody penetration and then washed in HS-PBS three times for 10 min each. Sections were then incubated at 37 °C for 30 min in HS-PBS and then for 15 min in 0.001% trypsin + 0.001% CaCl_2_ at 37 °C. Slides were washed in HS-PBS three times for 10 min each and incubated with primary antibodies diluted in HS-PBS with 0.3% Triton X-100 (HS-PBST) for 48 h at 4 °C. After 48 h, sections were incubated with primary antibodies for 30 min at RT. Sections were then washed in HS-PBS for 10 min six times and incubated with secondary antibodies diluted in HS-PBST for 2 h at RT or overnight at 4 °C. After secondary antibody incubation, sections were washed in HS-PBST three times for 10 min each and mounted with Fluoromount Aqueous Mounting Medium with DAPI. The following primary antibodies were used: guinea pig anti-VGLUT1 (Millipore, AB5905, 1:1,000), mouse anti-NLGN2 (Synaptic Systems, 129 511, 1:250), rabbit anti-GABRB3 (Biomatik, 1:500)^22^, rabbit anti-MECP2 (Michael Greenberg, 1:1,000), guinea pig anti-VGAT (Synaptic Systems, 131 004, 1:1,000), mouse anti-glycine receptor α1 (Synaptic Systems, 146 111, 1:500), mouse anti-gephyrin (Synaptic Systems, 147 111, 1:500), mouse anti-NeuN (Sigma, MAB377, 1:500) and goat anti-mCherry (Sicgen, AB0040, 1:500). Secondary antibodies used in this study were goat anti-guinea pig 488 (Thermo Fisher, A-11073), goat anti-mouse IgG1 647 (Thermo Fisher, A-21240), goat anti-mouse IgG1 488 (Thermo Fisher, A-21121), goat anti-rabbit 647 (Thermo Fisher, A-21245), goat anti-rabbit 488 (Thermo Fisher, A-11008), goat anti-chicken 488 (Thermo Fisher, A-21449) and donkey anti-goat 546 (Thermo Fisher, A-11056). All secondary antibodies were used at a 1:500 dilution. In some experiments, the secondary antibody solution contained IB4 (Isolectin GS-IB4) Alexa 647 conjugate (Invitrogen, I32450) at a 1:500 dilution.

For P0 glabrous forepaw and back hairy skin stains, pups were deeply anesthetized in an ice bath for several minutes before killing. Mice were decapitated, and tissue samples were collected and drop-fixed in Zamboni fixative for 2 h at 4 °C. The tissue was then washed several times over 1 h in PBS. Tissue was cryoprotected in 30% sucrose in PBS for two nights at 4 °C, embedded in Tissue-Tek OCT Compound (Sakura, 4583) and frozen over a dry ice and ethanol slurry. Tissue was stored at –80 °C until transverse sections at 30 µm were acquired using a cryostat, and slides were allowed to dry overnight at RT before staining or were stored at –20 °C until staining. Staining was performed using the 5% normal goat serum protocol as described above. The following primary antibodies were used: rabbit anti-GFP (Abcam, ab6556, 1:500) and chicken anti-NFH (Aves Labs, NFH, 1:1,000).

For immunohistochemistry following electrophysiological assays on 200-µm sagittal spinal cord slices, samples were incubated in 4% PFA for 30 min, washed several times in PBS and incubated overnight in IB4 in blocking solution (0.1% Triton X-100 and 5% normal goat serum) at 4 °C. Sections were then washed three times for 5 min each in PBS and mounted on slides with Fluoromount Aqueous Mounting Medium with DAPI.

### Puncta analysis

For puncta analyses, *z*-stack images of spinal cord slices were acquired on a Zeiss LSM 700 or LSM 900 confocal microscope using a ×63 oil immersion lens (Zeiss Plan-Apochromat ×63/1.40-NA). For neonatal versus adult immunohistochemistry imaging, blinding was not possible due to obvious differences in the size of samples between conditions. For control versus mutant imaging, the experimenter was blind to the conditions in most cases. Images were acquired in laminae III–IV of the dorsal horn, which, in some experiments, was identified by immunostaining with IB4 to delineate lamina II_iv_. The depth of the *z* stack was determined by the expanse of VGLUT1^+^ or VGAT^+^ terminals. Imaging parameters were held constant across samples of each slide. In most cases, contrast-limited adaptive histogram equalization was applied to the images, and cross-correlation analyses were performed using custom MATLAB code to ensure comparability across experiments and conditions. For puncta analyses, the experimenter was not blinded to the conditions of the experiments. Puncta analysis was performed in NIH ImageJ as described previously^22^. A custom script created a mask of VGLUT1^+^ or VGAT^+^ terminals >0.5 µm (VGLUT1^+^) or >0.1 µm (VGAT^+^) in diameter and performed thresholding on GABRB3, glycine receptor subunit α1, NLGN2 or gephyrin. Puncta of at least 0.1 µm in diameter that were contained within the VGLUT1 or VGAT mask were counted. Colocalization, or proximity values, were analyzed per 0.45- to 0.9-µm section thickness of tissue. Proximity values are reported, for example, for GABRB3 as GABRB3^+^VGLUT1^+^ puncta per total number of VGLUT1^+^ puncta per image. Three images were acquired per animal, and an average value was calculated per animal, which corresponds to each data point. The same parameters were used across tissues from the same slide. We note that the values derived from this method of analysis likely overestimate true synaptic colocalization/apposition due to the diffraction limit of confocal microscopy/objectives used as well as the size of optical sections (0.45 to 0.9 µm; thus receptor puncta at the bottom of an optical section may be spuriously notated as colocalized to a synaptic terminal marker that lies at the top of the section). We chose to use puncta contained within the VGLUT1/VGAT masks for both colocalization (within a presynaptic terminal) and apposition (a marker in the presynaptic terminal opposed to a marker in the postsynaptic specialization) because of the diffraction-limited nature of the microscopy method used (rather than analyze puncta contained within a defined radius of the VGLUT/VGAT masks, which would likely compound overestimation). To capture accurate colocalization/apposition values, additional deconvolution methods or high-resolution imaging techniques, such as array tomography, superresolution imaging or electron microscopy, would be warranted.

### In situ hybridization

Detection of *Nlgn2*, *Nefh* and *Calca* transcripts was performed by fluorescence in situ hybridization. Adult (>8-week-old) animals were killed by asphyxiation followed by cervical dislocation, and individual DRG were rapidly dissected from mice, frozen in dry ice-cooled 2-methylbutane and stored at –80 °C until further processing. The DRG were cryosectioned at a thickness of 20 µm, and RNA was detected using the RNAscope Fluorescent Multiplex Assay (ACD Bio), according to the manufacturer’s protocol. The following probes were used: *Mm-Nlgn2* exons 3–5 (made to order, 300031), *Mm-Nefh* (443671) and *Mm-Mrgprd* (417921). Sections were mounted in Fluoromount Aqueous Mounting Medium (Thermo Fisher, 00-4958-02) and imaged on a Zeiss LSM 700 confocal microscope.

### Dorsal column and dorsal column nuclei injections

For PSDC labeling experiments for electrophysiology in mature animals, P14–P16 C57BL/6J male and female mice were anesthetized by isofluorane inhalation (1.5–2.5%). Breathing rate and anesthesia were continuously monitored, and isofluorane levels were adjusted as necessary. The back of the neck was shaved and swabbed with betadine and 70% ethanol. A 5-mm incision was made in the skin, and 0.5% lidocaine was applied to the incision site and underlying muscle. Muscles were separated or cut to expose the cervical vertebral column. The dura and arachnoid membranes between C1 and C2 were cut to expose the spinal cord. Six 50-nl injections of CTB (recombinant) and Alexa Fluor 555 conjugate (Fisher, C34776) mixed with a nominal volume of fast green were made bilaterally into the dorsal column under visual guidance with a glass pipette (three on each side) at a rate of 50 nl min^–1^ using a Microinject system (World Precision Instruments). Injections were assessed by determining the extent to which the dorsal column took up the tracer, and an additional injection site was added when inefficient uptake was noted. Muscle and skin were then stitched together with sutures, and a liquid bandage (Nuskin) was applied. Before surgery, Nuskin was applied to the tail of the mother of experimental animals for habituation purposes. Buprenorphine was injected intraperitoneally for analgesia, and mice were returned to their home cage after recovery from anesthesia. Mice were monitored daily following surgery until they were killed for electrophysiology experiments 3–5 days after injection.

For PSDC labeling for P4 electrophysiological recordings, P3 C57BL/6J animals were initially anesthetized in an ice bath, and hypothermia was maintained on a cold plate during the surgery. A 3-mm incision was made in the skin, and 0.5% lidocaine was applied to the incision site and underlying muscle. Muscles were separated to expose the brainstem. The dura and arachnoid membranes were cut to expose the gracile and cuneate nuclei of the brainstem, and four to eight 10-nl injections were made bilaterally at a rate of 100 nl min^–1^. Surgery was performed in under 10 min. Following surgery, mice were transferred to a sanitized, ventilated and temperature-controlled 3 × 3 × 3 cm heated chamber (Warner, Dual Channel Temperature Controller, TC-344C) maintained at 34 °C with nesting from the home cage to minimize stress, and 4 mg per kg (body weight) carprofen was administered subcutaneously for analgesia. A cotton ball soaked with 3–5 ml of soymilk (Enfamil) was placed in the corner of the chamber, and animals were killed no more than 12 h following surgery for electrophysiological analyses.

### Spinal cord slice electrophysiological recordings

For electrophysiology in wild-type animals, C57BL/6J mice were used for each experiment. For mutant analyses, control littermates were used. For neonatal versus adult electrophysiology experiments, blinding was not possible due to obvious differences in the size of animals/samples between conditions. For control versus mutant experiments performed at the same age, the experimenter was blind to the conditions in most cases. P4 or P19–P21 mice were deeply anesthetized (using an ice bath or isofluorane, respectively) and rapidly transcardially perfused with RT choline chloride solution (92 mM choline chloride, 2.5 mM KCl, 1.2 mM NaH_2_PO_4_, 30 mM NaHCO_3_, 20 mM HEPES, 25 mM glucose, 5 mM sodium ascorbate, 2 mM thiourea, 3 mM sodium pyruvate, 10 mM MgSO_4_ and 0.5 mM CaCl_2_). Vertebral columns were dissected, and the lumbar spinal cord (L3–L6) was finely dissected in ice-cold choline chloride solution. Sagittal spinal cord sections (200 μm) were cut on a vibratome (Leica, VT1200S). Slices recovered for 30 min at 35 °C in artificial cerebrospinal fluid (aCSF) containing 2.0 mM CaCl_2_, 1 mM NaH_2_PO_4_, 119 mM NaCl, 2.5 mM KCl, 1.3 mM MgSO_4_, 26 mM NaHCO_3_, 25 mM dextrose and 1.3 mM sodium l-ascorbate (pH 7.4, 305–310 mOsm) oxygenated with 95% O_2_ and 5% CO_2_ for 30 min. For mIPSC recording experiments, recovery aCSF included 5 μM (*R*,*S*)-3-(2-carboxypiperazin-4-yl)propyl-1-phosphonic acid (CPP; Abcam, ab120160) and 5 μM 2,3-dihydroxy-6-nitro-7-sulfamoyl-benzo[f]quinoxaline (NBQX; Abcam, ab120046) to block excitatory glutamatergic activity in slices. For mEPSC recording experiments, recovery aCSF only included (*R*,*S*)-CPP to block NMDA receptors. After a recovery period, slices were kept at RT in the recovery chamber until recording experiments began.

Slices were placed in the recording chamber and secured using a harp (Warner Instruments, 64-1421). Cells were visualized using infrared differential interference contrast (DIC) microscopy or fluorescence microscopy, and recordings were performed in RT aCSF saturated with 95% O_2_ and 5% CO_2_ at a perfusion rate of 3–5 ml min^–1^. Whole-cell voltage clamp recordings from laminae III–IV or from CTB-labeled spinal cord neurons were obtained under visual guidance using a ×40 objective. Laminae III and IV were targeted based on distance from the substantia gelatinosa, which is readily visualized under infrared DIC. Following break-in, cells were allowed to dialyze for 5 min before data collection.

For mIPSC recording experiments, aCSF contained 5 μM (*R*,*S*)-CPP, 5 μM NBQX and 500 nM tetrodotoxin citrate (TTX; Tocris, 1069). For experiments in which GABAergic or glycinergic mIPSCs were isolated, 500 nM strychnine (Abcam, ab120416) or 5 μM SR 95531 (gabazine; Tocris, 1262), respectively, were either washed in or the entire recording was done with either drug in the aCSF. For mEPSC recording experiments, aCSF contained 5 μM (*R*,*S*)-CPP, 5 μM SR 95531, 500 nM strychnine and 500 nM TTX.

For mIPSC recordings, patch electrodes (3.0–4.5 MΩ) were filled with a CsCl-based internal solution containing 110 mM CsCl, 10 mM HEPES, 10 mM EGTA, 10 mM Cs-BAPTA, 4 mM CaCl_2_, 1 mM MgCl_2_, 10 mM TEA, 2 mM QX-314 and 0.2 mM D600 (pH 7.3, 295 mOsm), and neurons were voltage clamped at −60 mV. In some PSDC recording experiments, the internal solution also included ~100 µM Alexa Fluor 488 for cell dialyzation and subsequent visualization. For mEPSC recordings, patch electrodes were filled with a Cs-gluconate-based internal solution containing 130 mM Cs-gluconate, 10 mM HEPES, 1 mM EGTA, 0.1 mM CaCl_2_, 5 mM TEA, 1 mM QX-314 and 0.2 D600 (pH 7.3, 295 mOsm), and neurons were voltage clamped at −60 mV. Data were acquired using a Multiclamp amplifier, a Digidata 1440A acquisition system and pCLAMP10 software (Molecular Devices). The sampling rate was 20 kHz, and data were low-pass filtered at 3 kHz. No correction for junction potential was applied. Cells were discarded if residual uncompensated series resistance was >20 MΩ. Series resistance was monitored continuously throughout each experiment, and cells were excluded from analysis if these values changed by more than 20% during the experiment. For electrophysiology analyses, the experimenter was blinded to the experimental conditions. At least 100 events were analyzed per cell, which was performed in Clampfit (Molecular Devices).

### DRG electrophysiological recordings

P4 or P18–P30 mice were deeply anesthetized and rapidly transcardially perfused with choline chloride solution, as described above. The spinal column was removed, and four to six lumbar-level DRG were dissected and placed onto coverslips coated with poly-l-lysine (Sigma, P8920). The DRG were allowed to recover for 8 min (P4) or 15 min (P18–P30) in oxygenated aCSF solution at 35 °C containing 0.01–0.02% collagenase P (Sigma, 11213857001). RuBi-GABA (Fisher, 3400) stock solution was prepared by adding 10 μl of DMSO before adding water, as per the manufacturer’s instructions, to improve solubility. DRG were visualized using infrared DIC microscopy, and recordings were performed in RT oxygenated aCSF, as described above, with 100 μM RuBi-GABA. Recordings were performed in the dark, but computer monitors were not covered with filters. Patch electrodes (7.5–10.5 MΩ) were filled with a KCl-based internal solution containing 140 mM KCl, 10 mM HEPES and 0.5 mM EGTA (pH 7.3, 295 mOsm), and recordings were performed in the current clamp configuration. Only medium- to large-diameter cells (>35 μm in diameter and >55 pF capacitance)^57^ were analyzed with a resting membrane voltage of –60 to –70 mV, and cells were discarded if resting membrane voltage became higher than –55 mV during the recording. Cells were dialyzed for 5 min after break-in, and excitability and action potential waveforms were first assessed using increasing steps of current (100 pA). To perform uncaging experiments, LED whole-field illumination was used through a water immersion ×40 objective, and maximum depolarization in response to a 5-ms uncaging pulse (473 nM, ~5 mW) was recorded. Uncaging was performed three times per cell with 1 min in between each trial. The peak depolarization in a 10-ms window following the light pulse was analyzed, and the average of all three traces is reported per animal. No differences were found between C57BL/6J and *Gabrb3*^fl^ or Cre^–^ animals with respect to DRG depolarization responses to GABA or intrinsic excitability.

### Statistics and reproducibility

For all figures, data are expressed as the mean ± s.e.m., unless noted otherwise. For behavioral experiments, the number of animals per group used in each experiment are shown as individual data points in a graph. All behavioral, electrophysiology and immunohistochemistry experiments were conducted using two or more cohorts of animals. Power analysis statistical tests were used to approximate sample sizes for all behavioral and electrophysiology experiments. No statistical methods were used to predetermine sample sizes for immunohistochemical experiments. For images in Fig. 4a,b and Extended Data Figs. 2c and 3a, each experiment was repeated independently two or more times using different animals.

All datasets were tested for normality using a Shapiro–Wilk test, and Bartlett’s test for equal variance was applied to all datasets. Comparisons between groups in all experiments were performed using unpaired, two-tailed or one-tailed Student’s *t*-tests (in the case of two groups distributed normally), Welch’s *t*-tests (in the case of two groups distributed normally but with unequal variances), Mann–Whitney *U*-tests (for non-parametric data), one-way analysis of variance (in the case of three or more groups distributed normally), mixed-effect analyses (in the case of repeated-measures data from two groups with multiple conditions or time points where there were missing values), Kruskal–Wallis test (non-parametric test in the case of three or more groups) or one-sided Fisher’s exact test (for comparing categorical variables). One-tailed analyses were used to specifically test for the presence of overreactivity and a lack of sensory habituation in neonatal groups. Comparisons between groups with significant differences are indicated above the appropriate groups. Asterisks indicate statistically significant *P* values for Student’s *t-*tests, unless otherwise noted in the figure legends. For one-way analysis of variance, post hoc comparisons were performed using the post hoc test indicated in the figure legend. Statistically significant *P* values of post hoc comparisons are represented with asterisks in figures; ^#^*P* < 0.1; *P < 0.05; **P < 0.001; ***P < 0.0001. Statistical information can be found in Supplementary Table 1. All statistics were performed using GraphPad Prism.

### Reporting summary

Further information on research design is available in the Nature Portfolio Reporting Summary linked to this article.

## Online content

Any methods, additional references, Nature Portfolio reporting summaries, source data, extended data, supplementary information, acknowledgements, peer review information; details of author contributions and competing interests; and statements of data and code availability are available at 10.1038/s41593-023-01552-9.

### Supplementary information

Reporting Summary

Supplementary Table 1 Statistical information.

Supplementary Table 2 Genotyping primers used in this study.

Supplementary Video 1 Displacement responses to a 1.0-psi air puff stimulus in control and mutant animals at P4.

Supplementary Video 2 Displacement responses to activation of LTMRs in control and mutant E18.5 animals.

### Source data

Source Data Figs. 1–7 Statistical source data.

## Data Availability

Source data are provided with this paper. Other data and material are available from the corresponding author upon request.

## Code availability

The code for optical flow point tracking is available at https://github.com/RichieHakim/face-rhythm. Further code is available upon request.
