# Supplementary material for: The developmental timing of spinal touch processing alterations predicts behavioral changes in genetic mouse models of autism spectrum disorders
Source: Nat Neurosci. 2024 Jan 17;27(3):484–96. doi: 10.1038/s41593-023-01552-9 (PMC10917678; doi:10.1038/s41593-023-01552-9)
Supplement: Supplementary file 1 — Reporting Summary [file 41593_2023_1552_MOESM1_ESM.pdf]

nature portfolio

Corresponding author(s): David D. Ginty

Last updated by author(s): Nov 20, 2023

Reporting Summary

Nature Portfolio wishes to improve the reproducibility of the work that we publish. This form provides structure for consistency and transparency in reporting. For further information on Nature Portfolio policies, see our Editorial Policies and the Editorial Policy Checklist.

Statistics

For all statistical analyses, confirm that the following items are present in the figure legend, table legend, main text, or Methods section.

n/a	Confirmed	
☐	☒	The exact sample size (*n*) for each experimental group/condition, given as a discrete number and unit of measurement
☐	☒	A statement on whether measurements were taken from distinct samples or whether the same sample was measured repeatedly
☐	☒	The statistical test(s) used AND whether they are one- or two-sided<br>*Only common tests should be described solely by name; describe more complex techniques in the Methods section.*
☒	☐	A description of all covariates tested
☐	☒	A description of any assumptions or corrections, such as tests of normality and adjustment for multiple comparisons
☐	☒	A full description of the statistical parameters including central tendency (e.g. means) or other basic estimates (e.g. regression coefficient) AND variation (e.g. standard deviation) or associated estimates of uncertainty (e.g. confidence intervals)
☐	☒	For null hypothesis testing, the test statistic (e.g. *F*, *t*, *r*) with confidence intervals, effect sizes, degrees of freedom and *P* value noted<br>*Give P values as exact values whenever suitable.*
☒	☐	For Bayesian analysis, information on the choice of priors and Markov chain Monte Carlo settings
☒	☐	For hierarchical and complex designs, identification of the appropriate level for tests and full reporting of outcomes
☒	☐	Estimates of effect sizes (e.g. Cohen's *d*, Pearson's *r*), indicating how they were calculated

Our web collection on statistics for biologists contains articles on many of the points above.

Software and code

Policy information about availability of computer code

Data collection

PPI data was collected using San Diego Instruments startle reflex system (SR-LAB Startle Response System) and software.
Adult open field, 3-chamber social interaction, and elevated plus maze video footage was collected using IC Capture 2.5.
Neonatal air puff behavior video footage was collected using Point Grey FlyCap2 2.13.3.61, and air puffs were delivered using San Diego Instruments startle reflex system (SR-LAB Startle Response System) software.
Electrophysiology data was collected using Clampex (pCLAMP 10) and MultiClamp 700B.
Confocal images were collected using ZEISS ZEN software (multiple versions).

Data analysis

Adult behavior was analyzed using custom MATLAB scripts.
Neonatal behavior was analyzed using custom Python scripts.
Electrophysiology data were analyzed in Clampfit (pCLAMP 10).
Confocal imaging data were analyzed in ImageJ.
Statistics were performed in Graphpad Prism 9.
Figure assembly was done using Adobe Illustrator.
The code for optical flow behavioral tracking is available at https://github.com/RichieHakim/face-rhythm.

For manuscripts utilizing custom algorithms or software that are central to the research but not yet described in published literature, software must be made available to editors and reviewers. We strongly encourage code deposition in a community repository (e.g. GitHub). See the Nature Portfolio guidelines for submitting code & software for further information.

Data

Policy information about availability of data

All manuscripts must include a data availability statement. This statement should provide the following information, where applicable:

- Accession codes, unique identifiers, or web links for publicly available datasets
- A description of any restrictions on data availability
- For clinical datasets or third party data, please ensure that the statement adheres to our policy

Source data are provided with this paper. Other data and material are available from the corresponding author upon request.

Research involving human participants, their data, or biological material

Policy information about studies with human participants or human data. See also policy information about sex, gender (identity/presentation), and sexual orientation and race, ethnicity and racism.

Reporting on sex and gender	N/A
Reporting on race, ethnicity, or other socially relevant groupings	N/A
Population characteristics	N/A
Recruitment	N/A
Ethics oversight	N/A

Note that full information on the approval of the study protocol must also be provided in the manuscript.

Field-specific reporting

Please select the one below that is the best fit for your research. If you are not sure, read the appropriate sections before making your selection.

☒ Life sciences ☐ Behavioural & social sciences ☐ Ecological, evolutionary & environmental sciences

For a reference copy of the document with all sections, see nature.com/documents/nr-reporting-summary-flat.pdf

Life sciences study design

All studies must disclose on these points even when the disclosure is negative.

Sample size	Power analysis tests were used to predetermine approximate sample sizes for all behavioral and electrophysiology experiments. No statistical methods were used to predetermine sample sizes for immunohistochemical experiments, but sample numbers were similar to analyses previously published by our lab (Zimmerman et al., 2019).
Data exclusions	For PPI, animals whose baseline movement was greater than 25% of their acoustic startle response were not included in analyses (i.e. these animals exhibited insufficient acoustic startle amplitudes).<br>For neonatal (E18.5, P0, P4) behavior, trials in which the animal exhibited movement during the 10 frame baseline collection period prior to the stimulus were discarded during analysis. For habituation analyses, only animals that exhibited >20% of baseline movement to the 1st presentation of the 1.0 stimulus were included for analysis.
Replication	All behavioral, electrophysiology, and immunohistochemistry experiments were conducted using two or more cohorts of animals. All replication attempts were successful.
Randomization	In this study, animals were allocated into experimental groups based on genotype or age, so randomization was not applicable. To control for behavioral variation, littermates were co-housed in all cases, and behavioral assays were always performed on littermate cohorts. Additionally, animals within the same experimental groups were maintained on the same genetic backgrounds. All animals for each experiment type were subjected to the same assays and conditions. For immunohistochemistry variation, animals for the same experiment were always processed on the same day, using the same reagents, and were collected on the same slides for staining. Imaging parameters were held constant across the same experiment. For electrophysiology, variation was minimized by using the same experimental conditions and solution recipes across experimental groups, and all samples underwent the same assays/stimuli within the same experimental groups. The only exception is that not all samples underwent drug wash-ins, and these samples were randomly allocated.
Blinding	For all behavioral assays, experiments and analyses were performed by investigators blinded to genotype. For neonatal vs. adult immunohistochemistry imaging, blinding was not possible to due obvious differences in the size of samples between conditions. For control vs. mutant imaging, the experimenter was blind to conditions in most cases. For puncta analyses, the experimenter was not blinded to the conditions of the experiments. For neonatal vs. adult electrophysiology experiments, blinding was not possible to due obvious differences in

the size of animals/samples between conditions. For control vs. mutant experiments performed at the same age, the experimenter was blind to conditions in most cases. For electrophysiology analyses, the experimenter was blinded to experimental conditions.

Reporting for specific materials, systems and methods

We require information from authors about some types of materials, experimental systems and methods used in many studies. Here, indicate whether each material, system or method listed is relevant to your study. If you are not sure if a list item applies to your research, read the appropriate section before selecting a response.

Materials & experimental systems

n/a	Involved in the study
☐	☒ Antibodies
☒	☐ Eukaryotic cell lines
☒	☐ Palaeontology and archaeology
☐	☒ Animals and other organisms
☒	☐ Clinical data
☒	☐ Dual use research of concern
☒	☐ Plants

Methods

n/a	Involved in the study
☒	☐ ChIP-seq
☒	☐ Flow cytometry
☒	☐ MRI-based neuroimaging

Antibodies

Antibodies used

Primaries used:
Guinea pig anti-VGLUT1 (Millipore, AB5905, 1:1000), mouse anti-NLGN2 (Synaptic Systems, 129 511, 1:250), rabbit anti-GABRB3 (custom generated by Biomatik, 1:500), rabbit anti-MeCP2 (gift from Michael Greenberg lab, 1:1000), guinea pig anti-VGAT (Synaptic Systems, 131 004, 1:1000), mouse anti-glycine receptor α1 (Synaptic Systems, 146 111, 1:500), mouse anti-gephyrin (Synaptic Systems, 147 111, 1:500), mouse anti-NeuN (Sigma, MAB377, 1:500), goat anti-mCherry (Sicgen, AB0040, 1:500), rabbit anti-GFP (Abcam, ab6556, 1:500), and chicken anti-NFH (Aves Labs, NFH, 1:1000).

Secondaries used:
Secondary antibodies used in this study were goat anti-guinea pig 488 (Thermo Fisher, A-11073), goat anti-mouse IgG1 647 (Thermo Fisher, A-21240), goat anti-mouse IgG1 488 (Thermo Fisher, A-21121), goat anti-rabbit 647 (Thermo Fisher, A-21245), goat anti-rabbit 488 (Thermo Fisher, A-11008), goat anti-chicken 488 (Thermo Fisher, A-21449), and donkey anti-goat 546 (Thermo Fisher, A-11056). All secondaries were used at a 1:500 dilution. In some experiments, the secondary antibody solution contained IB4 (Isolectin GS-IB4), Alexa 647 conjugate (Invitrogen, I32450) at 1:500 dilution.

Validation

Guinea pig anti-VGLUT1 validated by vendor using western blot (WB) in KO mice.
Mouse anti-NLGN2 validated by prior study (Li et al., 2017, WB) in KO animals and in this study (IHC) conditional KO mice.
Rabbit anti-GABRB3 validated in Zimmerman et al., 2019 (IHC) in conditional KO mice.
Rabbit anti-MeCP2 validated in multiple studies, including Orefice et al., 2016, 2019, and in this study (IHC) in KO mice.
Guinea pig anti-VGAT validated in multiple studies, including Saito et al., 2010 in KO mice, and Pei et al., 2015 in conditional KO mice.
Mouse anti-glycine receptor α1 has not been validated due to lack of a KO mouse, but this study, as well as Koch et al., 2012 show lack of staining in the spinal cord at early developmental stages, when glycinergic activity is functionally minimal.
Mouse anti-gephyrin validated in O'Sullivan et al., 2016 in conditional KO mice (IHC).
Mouse anti-NeuN validated by vendor and publications for use in FC, IC, IF, IH, IH(P), IP and WB across species, including mouse.
Goat anti-mCherry validated by vendor using WB in non-mCherry expressing mouse tissues and cells.
Rabbit anti-GFP validated in several studies using mouse tissue, including in this study, where GFP signal is not visible in non-genetically labeled mouse tissues.
Chicken anti-NFH validated in Neubarth et al., 2020, where TrkB conditional knockout animals lose expression of NFH+ fibers in the skin.

Animals and other research organisms

Policy information about studies involving animals; ARRIVE guidelines recommended for reporting animal research, and Sex and Gender in Research

Laboratory animals

Male and female mice of mixed genetic backgrounds (C57BL/6J, 129/SvEv, CD1) were used for these studies. The only exceptions were Nlgn2 and Mecp2 germline mutant mice, which were backcrossed for at least 5 generations to a C57BL/6J background. Animals were co-housed in cages of 2-5 animals. Housing and testing conditions were maintained at 70-73°F (~21-22.8°C) with 40-60% humidity. For adult behavioral testing, mice were weaned and ear notched for genotyping at P21 (+/- 2 days), and testing was done beginning at 6 weeks of age and complete by 8 weeks of age. For embryonic and neonatal behavior, mice were toe-clipped following testing and genotyped. For electrophysiology experiments, mice were toe-clipped and genotyped prior to P4.

Wild animals

This study did not involve wild animals.

Reporting on sex

Male and female mice were used for all analyses, and no differences between male and female mice of the same genotype were observed. The only exception is that only hemizygous animals were used to study Mecp2 (males), and they were compared to male control littermates (Orefice et al., 2016; Orefice et al., 2019).

Field-collected samples	This study did not involve field-collected samples.
Ethics oversight	All procedures performed in this study were approved by the Harvard Medical School Institutional Animal Care and Use Committee (IACUC) and were performed in compliance with the Guide for Animal Care and Use of Laboratory Animals.

Note that full information on the approval of the study protocol must also be provided in the manuscript.
